# Defect Passivation in Lead‐Halide Perovskite Nanocrystals and Thin Films: Toward Efficient LEDs and Solar Cells

**DOI:** 10.1002/anie.202102360

**Published:** 2021-05-28

**Authors:** Junzhi Ye, Mahdi Malekshahi Byranvand, Clara Otero Martínez, Robert L. Z. Hoye, Michael Saliba, Lakshminarayana Polavarapu

**Affiliations:** ^1^ Cavendish Laboratory University of Cambridge 19, JJ Thomson Avenue Cambridge CB3 0HE UK; ^2^ Institute for Photovoltaics (ipv) University of Stuttgart Pfaffenwaldring 47 70569 Stuttgart Germany; ^3^ Helmholtz Young Investigator Group FRONTRUNNER IEK5-Photovoltaik Forschungszentrum Jülich 52425 Jülich Germany; ^4^ Department of Materials Imperial College London Exhibition Road London SW7 2AZ UK; ^5^ CINBIO Universidade de Vigo Materials Chemistry and Physics Group Department of Physical Chemistry Campus Universitario Lagoas, Marcosende 36310 Vigo Spain

**Keywords:** defect passivation, lead-halide perovskites, perovskite nanocrystals, perovskite solar cells, surface chemistry

## Abstract

Lead‐halide perovskites (LHPs), in the form of both colloidal nanocrystals (NCs) and thin films, have emerged over the past decade as leading candidates for next‐generation, efficient light‐emitting diodes (LEDs) and solar cells. Owing to their high photoluminescence quantum yields (PLQYs), LHPs efficiently convert injected charge carriers into light and vice versa. However, despite the defect‐tolerance of LHPs, defects at the surface of colloidal NCs and grain boundaries in thin films play a critical role in charge‐carrier transport and nonradiative recombination, which lowers the PLQYs, device efficiency, and stability. Therefore, understanding the defects that play a key role in limiting performance, and developing effective passivation routes are critical for achieving advances in performance. This Review presents the current understanding of defects in halide perovskites and their influence on the optical and charge‐carrier transport properties. Passivation strategies toward improving the efficiencies of perovskite‐based LEDs and solar cells are also discussed.

## Graphical abstract

Despite the defect‐tolerance of lead‐halide perovskites, defects at the surface of colloidal nanocrystals and grain boundaries in thin films play a critical role in charge‐carrier transport and nonradiative recombination, which lowers the photoluminescence quantum yields, device efficiency, and stability. This Review summarizes the defects, their influence on the optical and charge‐carrier transport properties, and passivation strategies to mitigate the effects of defects.

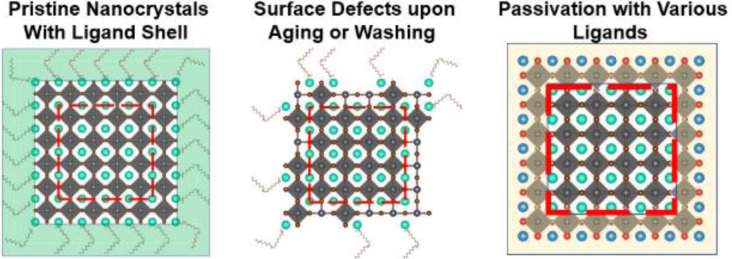

## 1 Introduction

Defects exist in nearly all semiconductors at room temperature.^[1]^ These defects exist at the atomic scale (e.g. vacancies) through to the macroscopic level (e.g. voids or wrinkles).[^1c^, ^2^] They play a key role in controlling the optical, electronic, and structural properties of semiconductors, and thus affect a wide range of applications.[^1a^, ^1d^, ^1f^, ^2a^, ^3^] Although defects give rise to numerous advantages (e.g. active sites for photocatalysis), the traps they introduce in the band gap are detrimental to the optoelectronic performance of semiconductors.[^1a^, ^1g^] Historically, the emphasis of material growers was to develop synthesis routes to prepare pure and perfect crystalline semiconductors which could be controllably doped with the desired elements to obtain the required electronic properties (e.g. majority carrier type and concentration).[^1a^, ^4^] The presence of defects in certain semiconductors, even at a level of parts per billion, can dramatically alter their properties, such as conductivity, charge‐carrier mobility, and minority carrier lifetimes. Such defects can, therefore, lower the performance of the resulting optoelectronic devices.

In classical semiconductor systems, such as Si, GaAs, CdTe, CdSe, InP, and metal chalcogenides, a small population of defects can be extremely detrimental, as reflected in the substantial decrease in their intrinsic photoluminescence quantum yield (PLQY), carrier mobility, and stability.[^1f^, ^5^] However, interestingly, some semiconductors are defect‐tolerant, which means that it is possible to achieve low nonradiative recombination rates despite high densities of traps in the band gap.[^1a^, ^2c^, ^6^] Defect‐tolerant semiconductors were rare and were not widely studied until the recent serendipitous discovery of LHPs.^[7]^ LHPs have the general formula ABX_3_, where A=CH_3_NH_3_^+^, HC(NH_2_)_2_^+^, Cs^+^; B=Pb^2+^; X=Cl^−^, Br^−^, and I^−^. This class of halide perovskites shot to prominence over the past decade owing to the rapid increases in efficiency achieved in optoelectronic devices. Originally, this was achieved in photovoltaic devices, whose efficiency rose from 3.8 % in 2009^[8]^ to a certified 25.5 % in 2020.^[9]^ Subsequently, efficient performance was also found in LEDs (from ca. 1 % external quantum efficiency in 2014 to >23 % in 2020^[10]^), radiation detectors, and others.^[11]^

Nevertheless, despite their defect‐tolerant nature, the surface defects and grain boundaries (GBs) present in perovskite thin films have been found to be detrimental to the performance of the resulting optoelectronic devices.[^2b^, ^2c^, ^2d^, ^6c^, ^12^] The latest advances in efficiency require careful passivation strategies.^[13]^ Similarly, in colloidal perovskite NCs, the surface defects arising from the removal of ligands and surface halides during purification significantly reduce the PLQY.^[14]^ In particular, surface defects play a critical role in the optical properties of colloidal NCs because of their high surface area to volume ratio.[^14^, ^15^] Therefore, it is of utmost importance to understand the types of defects that are common at surfaces as well as their role in the optoelectronic properties of perovskite thin films and colloidal NCs to advance their applications in solar cells and LEDs.[^2b^, ^12a^, ^14c^, ^16^] Toward this goal, a wide range of surface passivation strategies have been developed using a large variety of organic and inorganic molecules for LHP NCs as well as thin films.[^2b^, ^12a^, ^14b^, ^14c^, ^16a^, ^17^] In this Review, first we present a fundamental understanding of the surface chemistry and defects in perovskite NCs and thin films. The defect‐tolerance of different compositions of LHPs is discussed, as well as the types of defects that commonly occur. We then provide an overview of surface passivation strategies for both colloidal NCs and thin films toward improving their optical properties and thus the efficiencies of the resulting LEDs and solar cells. Finally, we present a brief outlook, highlighting the open questions and remaining challenges in this research field.

## 2 Surface Chemistry and Defects in Thin‐Film and Colloidal Perovskites

As discussed in the introduction, surface chemistry plays a critical role in the optical and electronic properties as well as the stability of LHPs. Therefore, the surface chemistry of LHPs has been heavily investigated but is still not fully understood.[^15^, ^16^, ^18^] During the crystallization of perovskite thin films, their surfaces can terminate with PbX_2_ or AX or both, depending on the film processing conditions. The electronic band structure of the surface could be different from that of the bulk, and the type of surface termination can significantly influence the band alignment with other molecular systems.^[19]^ Although AX termination is thermodynamically stable, it can be changed to PbX_2_ when organic‐inorganic hybrid perovskite films are exposed to moisture because of differences in their interaction with water molecules. This is because the AX termination can undergo fast hydration compared to the PbX_2_ termination because of the strong Pb−X bond.

**Figure 1:**
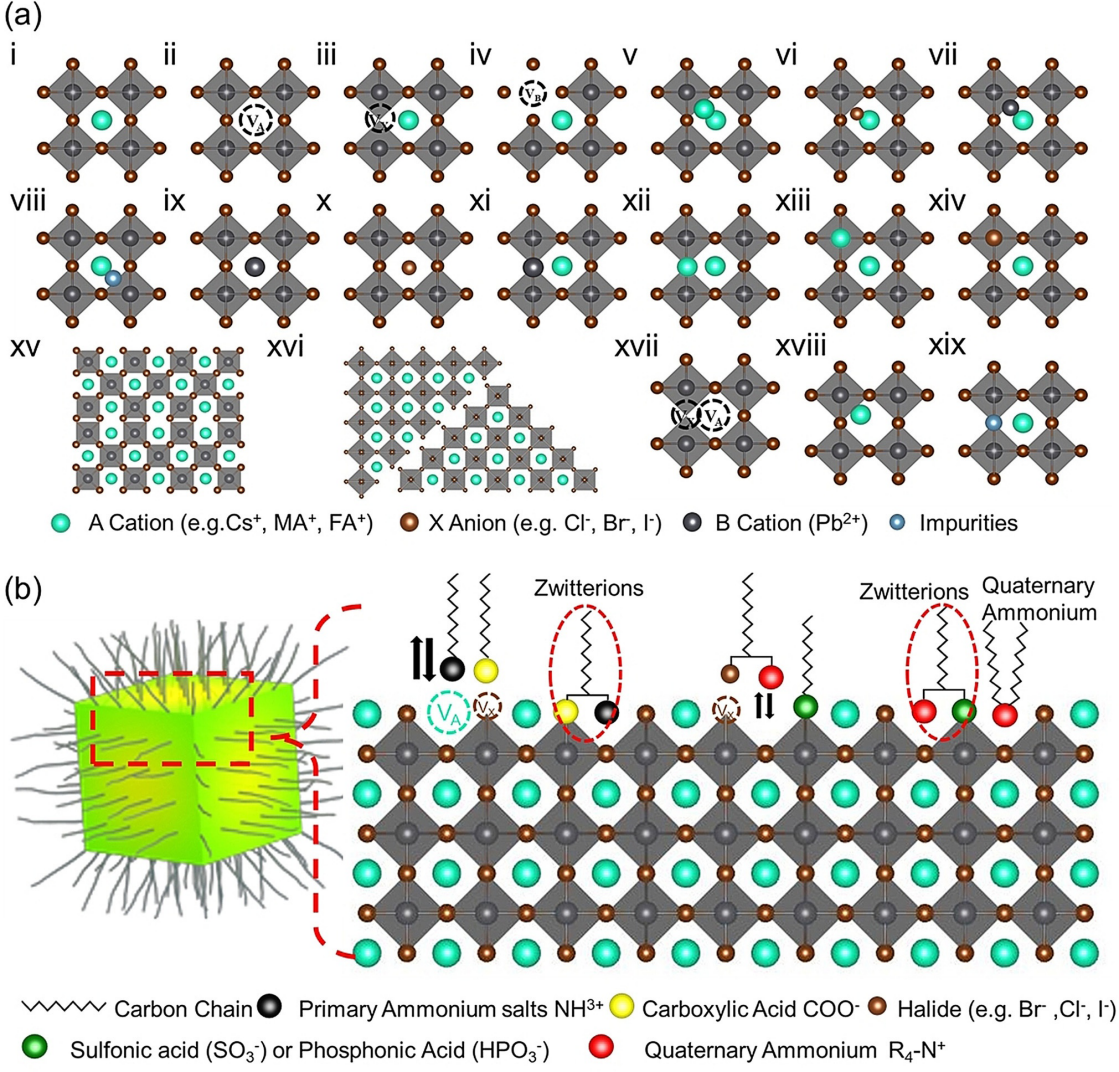
Defects in lead‐halide perovskites. a) Types of defects in lead‐halide perovskites: i) perfect crystals, ii) A‐site vacancy, iii) X‐site vacancy, iv) B‐site vacancy, v) A‐site interstitial, vi) X‐site interstitial, vii) B‐site interstitial, viii) impurity interstitial, ix) A_B_ anti‐sites, x) A_X_ anti‐sites, xi) X_B_ anti‐sites, xii) X_A_ anti‐sites, xiii) B_A_ anti‐sites, xiv) B_x_ anti‐sites, xv) lattice dislocations, xvi) grain boundary defects, xvii) Schottky defect (anion and cation vacancies occurring together), xviii) Frenkel defect (interstitial and vacancy created from the same ion), xix) impurity substitution. b) Schematic illustration of the ligand‐capped LHP nanocrystal and an enlarged view of the nanocrystal surface with defects and different types of surface ligands used for the stabilization of NCs, including alkylammonium ions, carboxylic acid, and zwitterions such as amino acids. The Figure is inspired by Ref. [21].

However, the presence of higher humidity can lead to complete hydration of the surface, regardless of the type of surface termination. To improve the stability of LHP films by increasing the surface resistance to moisture, long‐chain alkylammonium halide molecules have been incorporated into perovskite films, thereby giving rise to 2D halide perovskites.^[20]^ These molecules can also passivate the surface defects in LHP films. Like in classical semiconductors, two of the main classes of defects are point defects (atomic‐scale) and structural defects. Compared with a perfect lattice (Figure 1 a, i), point defects (native defects) include vacancies (atoms absent from their lattice sites; Figure 1 a, ii–iv), interstitial defects (atoms located between lattice sites; Figure 1 a, v–vii), and anti‐site defects (atoms occupying another species’ lattice site; Figure 1 a, ix–xiv). Structural defects (extended defects) include dislocations (Figure 1 a, xv) and GBs (Figure 1 a, xvi). In addition, defect complexes can occur. These include Schottky defects (pair of anion and cation vacancies; Figure 1 a, xvii) and Frenkel defects (a pair of an interstitial and a vacancy created from the same ion; Figure 1 a, xviii). Beyond these intrinsic defects, extrinsic impurities can also occur (Figure 1 a, viii, xix). Unlike conventional semiconductors such as Si, CdTe, or GaAs, which require low defect concentrations to mitigate the deleterious effects of their deep traps, the common point defects in perovskites (A‐ and X‐site vacancies) are usually shallow‐level defects (at least for Br‐ or I‐based perovskites) and are not detrimental to their device performance. Point defects with deep traps such as interstitial or anti‐site defects are almost absent in perovskites, since they have high formation energies.^[22]^ Therefore, the types of defect which we should consider during synthesis and passivation are more likely to be uncoordinated Pb^2+^ ions and surface charged defects.[^22^, ^23^] More details will be discussed in Section 2.1.

On the other hand, the surface chemistry of colloidal perovskite NCs is more complex in the presence of capping ligands on the surface (Figure 1 b).[^6e^, ^18^] Most often, long‐chain amines in combination with a long‐chain acid have been used as ligands in the synthesis of colloidal perovskite NCs. One of the most commonly used amine‐acid pair is oleylamine and oleic acid. In this base‐acid pair, the proton is exchanged from the acid to the amine, leading to the formation of an ammonium cation (R‐NH_3_^+^), which then occupies the A site positions on the NC surface (Figure 1 b). Theoretical studies suggest that ammonium cation ligands replace 50 % of the “A” cations on the NC surface and these ligands help to maintain the colloidal stability of perovskite NCs in nonpolar solvents.^[24]^ Furthermore, it was shown experimentally (by X‐ray photoelectron spectroscopy (XPS) and ^1^H NMR spectroscopy) that the R‐NH_3_^+^ binds to the surface of CsPbBr_3_ NCs by replacing most of the Cs^+^ cations and forming hydrogen bonds with Br^−^ anions.^[18b]^ However, interestingly, it was argued that the R‐COO^−^ ions do not bind to the NC surface, but play an important role in the colloidal stability of CsPbBr_3_ NCs by forming an equilibrium with surface‐bound R‐NH_3_^+^ ions and maintaining charge neutrality.^[18b]^ In contrast, quantitative ^1^H NMR studies by Smock et al. suggest that both the oleylamine and oleic acid ligands bind to the surface of CsPbBr_3_ NCs.^[18a]^ Similarly, De Roo et al. showed, by using ^1^H NMR spectroscopy, that oleic acid binds to the NC surface in the form of oleylammonium oleate only when excess oleylamine is added to the purified NC solution, and this helps to improve the PLQY.^[15]^ Another hypothesis is that the long‐chain carboxylic acid molecules replace some of the surface halides to complete the octahedral coordination of lead, and thus passivate the mid‐gap defect states (Figure 1 b).^[21]^ Amine‐free synthesis methods were also reported, in which the nanocrystals were solely capped by oleic acid;^[25]^ this results in colloidal CsPbBr_3_ NCs with only oleate‐terminated surfaces. Although the role of oleic acid in the colloidal stability of perovskite NCs is still under debate, it is widely accepted that alkylammonium cation ligands bind to the surface of perovskite NCs. However, ligand binding to a perovskite NC surface is highly dynamic and, therefore, the surface is properly passivated only in the presence of excess free ligands in the solution. Because they are labile, surface ligands often desorb from the perovskite NC surface upon ageing, dilution, or purification by washing, and these processes lead to the formation of surface defects, and thus a reduction in the PLQY.^[15]^ It is worth mentioning that a small amount of additional ligands need to be added during the purification to maintain the colloidal stability and high PLQY of perovskite NCs.^[15]^ To overcome these difficulties, various ligands, such as quaternary alkylammonium cations, alkyl phosphonates, and zwitterionic molecules, have been tested as alternatives to the oleylamine/oleic acid pair (Figures 1 b).^[26]^ It is well‐established that the stability and PLQY of perovskite NCs strongly depend on the binding affinity of the capping ligands. Recent studies suggest that ligands with multiple functional groups with opposite charges (e.g. zwitterionic molecules such as 3‐(*N*,*N*‐dimethyloctadecylammonio)propane sulfonate) tightly bind to the perovskite NC surface and thus significantly improve the stability. It was hypothesized that the ammonium cation of the zwitterionic molecules occupies the A‐site position, while the carboxylate or sulfonate group simultaneously binds to uncoordinated Pb to complete its octahedral coordination (Figure 1 b). In this regard, Krieg et al. demonstrated that the CsPbBr_3_ NCs capped with zwitterionic molecules could be washed multiple times without hampering the PLQY and chemical stability, thus suggesting that these types of ligands bind strongly to the perovskite NC surface.^[26g]^

In principle, perovskite NCs also exhibit similar types of defects as bulk thin films (Figure 1 a). Additionally, perovskite NCs exhibit surface vacancies caused by the desorption of ligand molecules (Figure 1 b). To address this, and owing to the flexible surface chemistry of perovskite NCs, a wide range of post‐synthetic defect‐passivation strategies have been developed to improve the stability and PLQYs, which is discussed in detail in Section 3. The defects in both thin films and NCs can cause either shallow or deep states in the electronic band structure. Defects in a specific material are generally studied by calculation of their formation energies in different charge states. It has been said that the vacancies (A, Pb, and X point defects) in perovskites form shallow traps because of their low formation energy. However, interestingly, surface halide vacancies can significantly affect nonradiative recombination rates depending on the type of halide, which are discussed in detail in the following section.

### 2.1 Defect Tolerance in Perovskites

**Figure 2:**
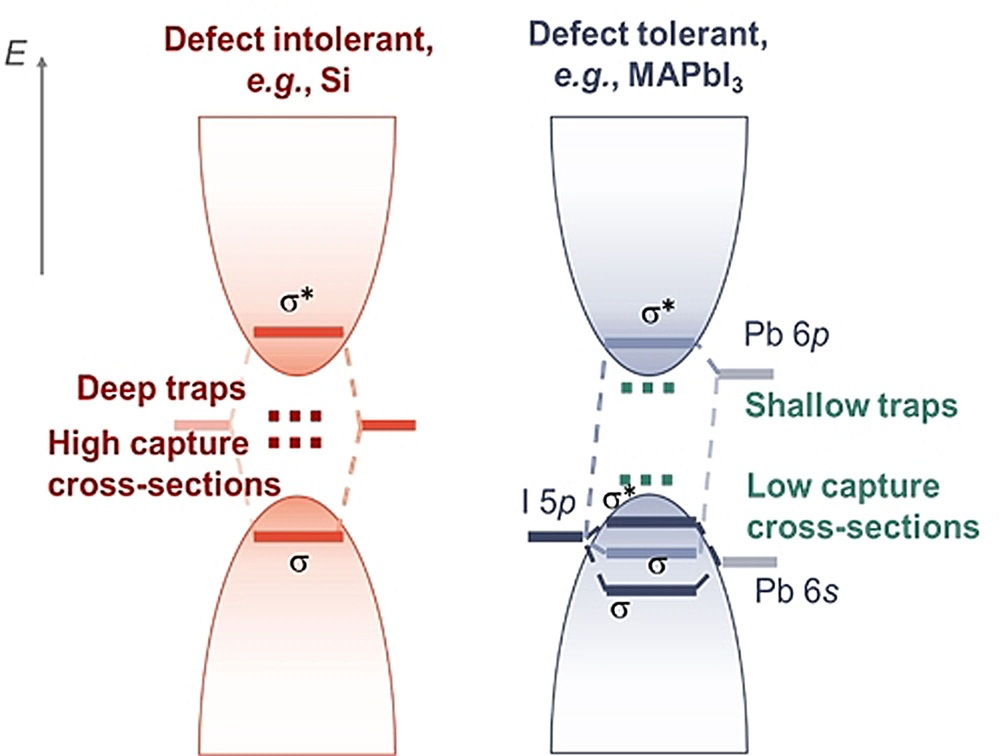
Illustration of how the electronic structure can enable defect tolerance. Left: The electronic structure of defect‐intolerant materials (e.g. silicon), in which traps primarily form deep within the band gap and have high capture cross‐sections. Right: the electronic structure of defect‐tolerant materials (e.g. MAPbI3), which primarily form shallow traps with low capture cross‐sections.

A key enabling feature of lead‐halide perovskites is their defect tolerance, in which low nonradiative recombination rates are achieved despite high densities of defects.[^6a^, ^6e^, ^27^] Owing to their defect tolerance, lead‐halide perovskite thin films and nanocrystals that have defect densities several orders of magnitude higher than silicon or GaAs could still realize efficient solar cells and light‐emitting diodes.[^22^, ^28^] This is because the trap levels from the most common defects are shallow and have low capture cross‐sections, and can be understood from the classic equation for Shockley‐Read‐Hall (*U*_SRH_) recombination [Eq. 1],^[6e]^ where *n* and *p* are the electron and hole carrier concentrations, respectively; *n*_1_ and *p*_1_ are the occupancy of the single trap‐state modelled; and *τ*_0,h_ and *τ*_0,e_ are the low injection hole and electron lifetimes, respectively.^[6e]^ Based on Equation (1), there are three ways to limit *U*_SRH_, as shown in Equations 2 and 3: 1) by having a low trap density (*N*_t_); 2) by having low carrier capture cross‐sections (*σ*_t,h_ and *σ*_t,e_); and 3) by having the defect energy levels (*E*_t_) far away from the map‐gap energy level (i.e. close to the band edge), thereby giving rise to a larger *n*_1_ or *p*_1_ value. In the third case, having a trap close to one band edge would allow it to be highly occupied by one of the carriers (hence a high *n*_1_ or *p*_1_ value) but it would be difficult for the other carrier to reach that trap state because of the large energy difference. Thus, nonradiative recombination is reduced. Traditionally, material growers have opted for the first option of minimizing *N*_t_. Defect tolerance focuses on designing materials that have low capture cross‐sections and shallow defects, enabling low *U*_SRH_ values despite high *N*_t_ values, as shown in Figure 2.

(1) $$U_{SRH}=\frac{np-n_{i}^{2}}{\tau_{0,h}\left(n+n_{1}\right)+\tau_{0,e}\left(p+p_{1}\right)}$$

(2) $$\tau_{0,h}=\frac{1}{N_{t}\sigma_{t,h}v_{th,h}}\;\;and\;\;\tau_{0,e}=\frac{1}{N_{t}\sigma_{t,e}v_{th,e}}$$

(3) $$n_{1}=n_{i}exp\left(\frac{E_{t}-E_{i}}{kT}\right)\;\;and\;\;p_{1}=n_{i}exp\left(\frac{E_{t}-E_{i}}{kT}\right)$$

**Figure 3:**
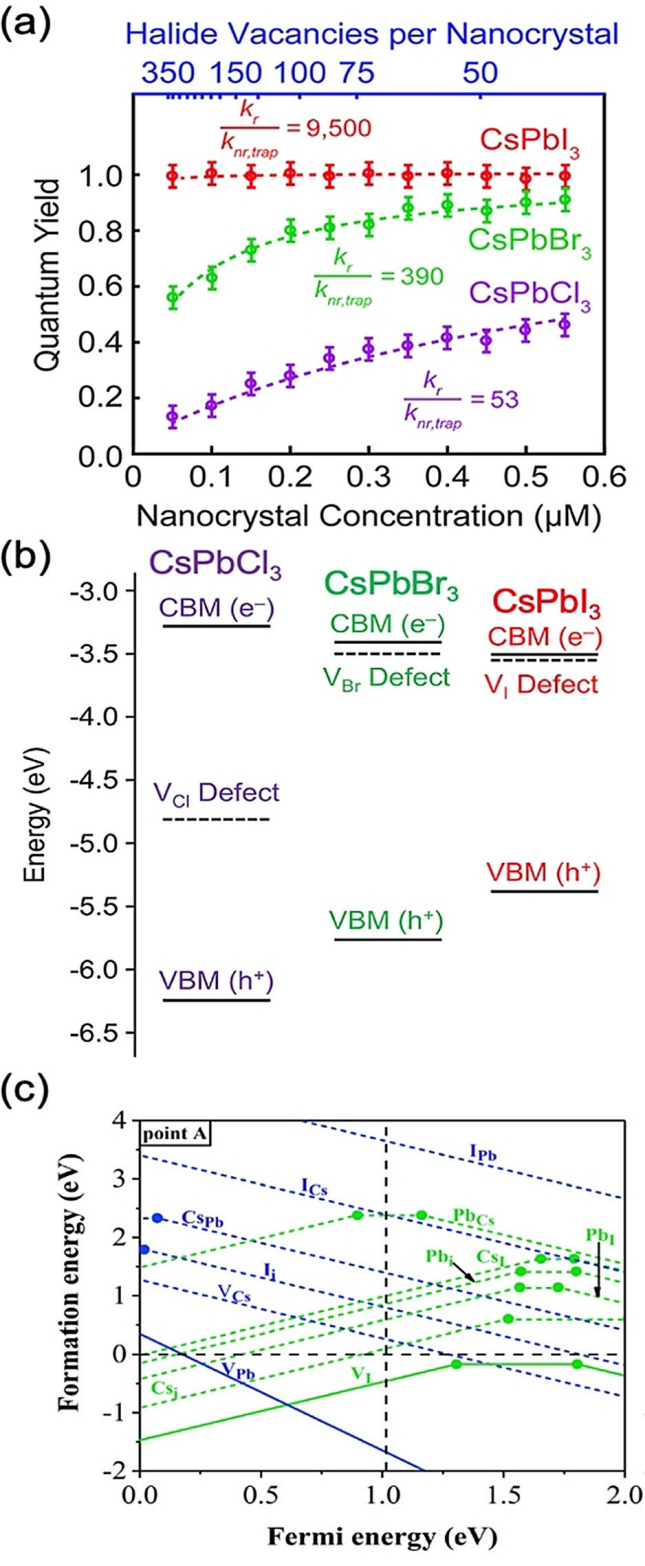
Defect tolerance in perovskite NCs. a) Effect of halide vacancies on the PLQYs of CsPbI_3_, CsPbBr_3_, and CsPbCl_3_ NCs, and b) transition level of halide vacancies in the band gap of these materials. Reprinted from Ref. [31] with permission. Copyright 2018, American Chemical Society. c) Defect diagram for CsPbI_3_. The dashed lines represent the conduction band minimum energies computed from GW calculations. Reprinted from Ref. [30b] with permission. Copyright 2017, American Institute of Physics.

Defect tolerance in the lead‐halide perovskite family was originally identified in methylammonium lead iodide (MAPbI_3_),^[29]^ and rationalized on the basis of the electronic structure.[^6b^, ^6e^] The Pb 6s and I 5p orbitals hybridize to form a pair of bonding and antibonding states in the upper valence band, while the Pb 6p orbital hybridizes with the I 5p orbital to form a bonding state in the upper valence band and an antibonding state in the conduction band minimum.^[6e]^ As a result, the original atomic orbitals are close to the band edges rather than inside the band gap (as shown in Figure 2, right). To a first approximation, defect states form close to the original atomic orbitals, and the electronic structure of MAPbI_3_ makes it more likely that these states are resonant within the bands or shallow within the band gap. This contrasts with the electronic structure of traditional semiconductors (e.g. silicon; Figure 2, left), in which the bonding–antibonding states form across the band gap and the original atomic orbitals are within the band gap, thereby making deep traps more likely to form. Furthermore, the presence of heavy elements in MAPbI_3_ leads to strong spin–orbit coupling, which reduces the band gap, making it more likely that the trap states are shallow.[^6b^, ^6e^] The high polarizability of the Pb^2+^ ion also leads to a high dielectric constant, which favors lower capture cross‐sections of charged defects.[^6e^, ^11^] The combination of shallowness in the trap energy levels and low capture cross‐sections leads to low trap‐assisted recombination rates despite high defect densities. However, the defect tolerance of MAPbI_3_ has not been found to be generalizable across the entire lead‐halide perovskite family (Figure 3 a). Beyond the electronic structure, the crystal structure has also been found to play an important role. For example, CsPbI_3_ can form three polymorphs (cubic α‐phase, or orthorhombic δ‐ and γ‐phases).^[30]^ Although the composition of the orbitals and bonding/antibonding states is qualitatively the same as that of MAPbI_3_, the Pb‐I‐Pb bond angle reduces from 180° for the α‐phase to 155° (γ‐phase) and 95° (δ‐phase).^[30a]^ Whereas α‐CsPbI_3_ is defect‐tolerant,^[30b]^ the reduced bond angles in the alternative polymorphs lead to a reduction in the overlap in the orbitals from Pb and I, thus reducing defect tolerance, especially in the δ‐phase, which forms deep traps.^[30a]^ Indeed, the synthesis of nanocrystals is advantageous because with reductions in size, formation of the α‐phase becomes more favorable, whereas the defect‐intolerant δ‐phase would form in bulk thin films or nanocrystals that are too large.^[32]^

Another important structural factor is the lattice parameter. On changing the halide from I^−^ to Br^−^ to Cl^−^ in MAPbX_3_, the lattice parameter decreases. This leads to stronger interactions between Pb^2+^ dangling bonds when halide vacancies form, which, when combined with the lower electron affinity, gives rise to deeper trap levels for the halide vacancy. Thus, while MAPbI_3_ and MAPbBr_3_ primarily form shallow traps, MAPbCl_3_ forms deep traps.[^14a^, ^23^, ^34^] This has also been found in their inorganic counterparts, in which cubic CsPbCl_3_ forms deep traps and has significantly lower PLQYs than α‐phase CsPbBr_3_ and CsPbI_3_, which are both defect‐tolerant (Figures 3 a,b).[^6d^, ^30b^, ^31^] Calculations of defects in α‐CsPbI_3_ showed the defects with the lowest formation energies are lead vacancies (V_Pb_), iodine vacancies (V_I_), as well as anti‐site (Pb_I_ and Cs_I_) and interstitial defects (Pb_i_ and Cs_i_). However, none of these defects have transition levels (or trap states) within the computed band gap.^[30b]^ We note, however, that the calculated band gap of about 1 eV from GW calculations is below the experimental band gap of 1.73 eV.^[35]^ Band‐gap corrections can lead to the transition levels also shifting in energy, but it is likely that the V_I_ transition level falls within the band gap, as has been found in computations by other groups (Figure 3 b). Similarly, calculations on defects in CsPbBr_3_ showed that all defects with low formation energies form shallow transition levels.^[6d]^

These discussions on the role of bulk point defects have been found to be applicable to surfaces, which play a critical role in nanocrystals. Ab initio calculations on CsPbX_3_ (X=I^−^, Br^−^, Cl^−^) slabs with a halide vacancy introduced on the surface show that the halide vacancy is shallow in CsPbI_3_, slightly deeper in CsPbBr_3_, and close to mid‐gap in CsPbCl_3_ (Figure 3 b). This is consistent with the trend in the PLQYs decreasing as the halide is changed from I to Cl, as well as the PLQY of CsPbI_3_ remaining close to unity as the halide vacancy content increased, whereas the PLQY of CsPbCl_3_ sharply decreased.^[31]^ However, ten Brinck et al. argued that understanding defects at the surfaces of nanocrystals computationally requires a different treatment to the bulk. Their work on CsPbBr_3_ nanocrystals, nevertheless, still showed that the surface is defect‐tolerant because the defects with the lowest formation energies remain shallow.^[14a]^

## 3 Defect Passivation in Perovskite NCs

**Figure 4:**
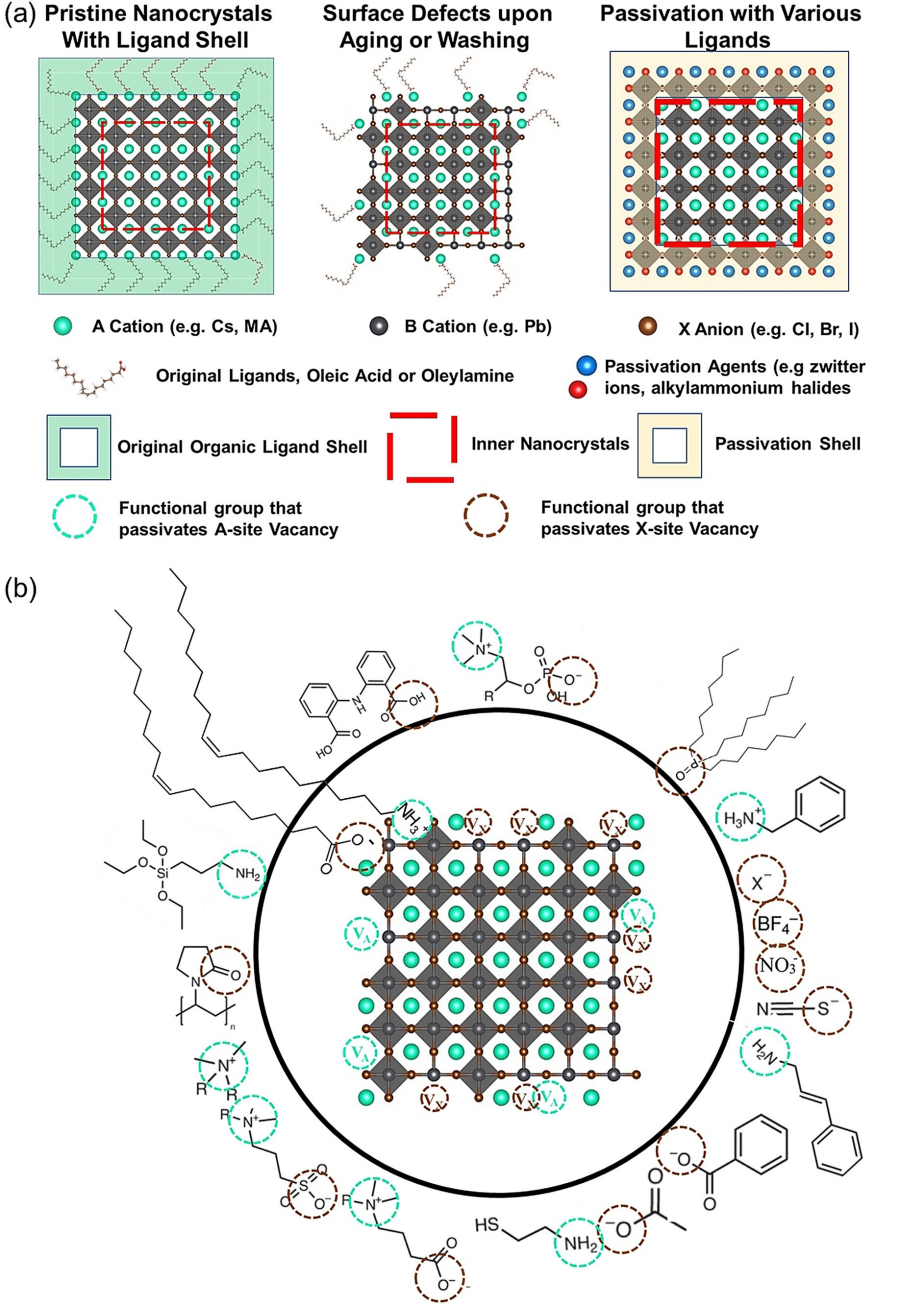
Surface chemistry of perovskite nanocrystals and defect passivation. a) Illustration of the formation of surface defects on pristine nanocrystals with well‐capped organic ligands. Aging or washing processes will lead to ligand detachment and surface defects. Surface passivation agents are used to reform the protective shell. b) Types of passivation ligands used in perovskite nanocrystal systems. The Figure is inspired by Refs. [17a, 33].

Figure 4 a illustrates the formation of surface defects in perovskite NCs either by ageing or washing, and their passivation with ligand molecules or metal salts. Ligand detachment from the NC surface leads to the formation of both A‐site and X‐site vacancies for charge neutralization (see Section 2 for more details). There are mainly three types of post‐synthetic approaches for the surface passivation of perovskite NCs: 1) addition of metal salts along with ligand molecules, 2) addition of ligand molecules that bind strongly to the perovskite NC surface, and 3) treatment with inorganic salts. The ligand molecules are expected to fill the A‐site and X‐site vacancies to recover the fluorescence of the perovskite NCs (Figure 4 a). According to covalent bond classification, these surface ligands are generally divided into L, X, and Z types depending on the number of electrons (2, 1, and 0, respectively) they donate to the metal of a NC surface. Because of the ionic nature of perovskite NCs, L‐type ligands (cationic and anionic) have mostly been used for the passivation of the A and X sites of the NC surface. One of the early studies on the surface passivation of LHP NCs was reported by Pan et al. 2015;^[36]^ they demonstrated an improvement in the photoluminescence and stability of CsPbBr_3_ NC films upon treatment with didodecyldimethylammonium sulfide (S^2−^‐DDA^+^). The authors reported that passivation leads to the formation of a protective layer enriched with sulfide. However, the passivation mechanism at the molecular level was unclear. The first detailed study of the surface chemistry of perovskite NCs and the highly dynamic nature of ligand binding was reported by Roo et al. in early 2016.^[15]^ They also found that the addition of excess amine improves the binding of carboxylic acid and thus the PLQY. Later, Pan et al. found that exchanging the ligands of the classical oleylamine/oleic acid pair by alkylammonium halides could significantly improve the PLQY and the efficiency of the resulting LEDs.^[37]^ Following these early studies, a large variety of ligands have been reported for the electronic passivation of perovskite NCs to improve their optical properties; some of the ligands are illustrated in Figure 4 b.[^14b^, ^14c^, ^14d^, ^26d^, ^31^, ^33^, ^38^] In most studies, it was shown that the passivating ligands help to improve the PLQY as well as the stability of perovskite NCs.

**Figure 5:**
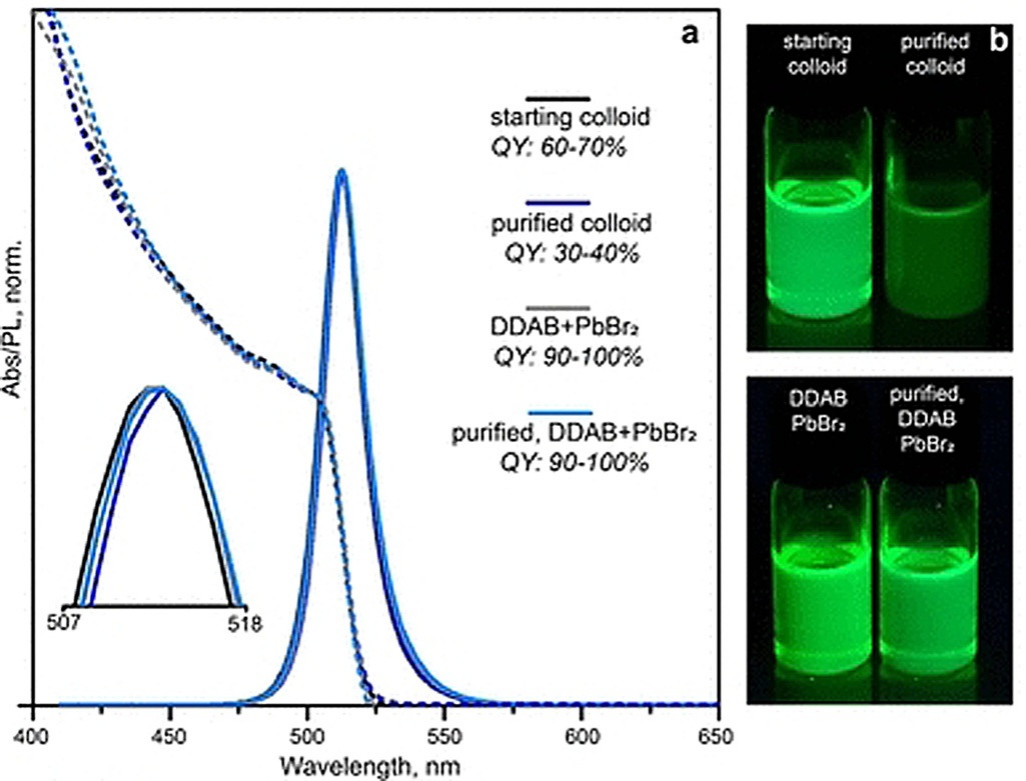
a) UV/Vis absorption and PL spectra of pristine and treated CsPbBr_3_ colloidal solutions: pristine oleylamine/oleic acid stabilized NCs (black line), colloidal solution purified using acetone as a nonsolvent (containing OLA and OA; dark blue line), pristine CsPbBr_3_ NCs treated with a mixture of DDAB+PbBr_2_ (gray line), and purified colloid treated with a mixture of DDAB+PbBr_2_ (blue line). The inset magnifies the PL band positions of the four samples; the PL position remains unaltered after surface treatment. b) Photographs of the four colloidal solutions under illumination with UV light. The Figures are reproduced from Ref. [17a] with permission under the Creative Common CC‐BY‐NC‐ND license. Copyright 2018, American Chemical Society.

The choice of the ligands is generally based on the simultaneous refilling of A‐site and halide vacancies with an ammonium cation and the same halide (or with an organic X‐type ligand), respectively[^14b^, ^17a^, ^18b^] This is because the healing of surface defects requires restoration of the damaged surface by filling the halide vacancies and forming a complete ligand shell. Keeping this in mind, alkylammonium halides have been a good choice for fulfilling this criterion toward effective electronic passivation and improving the stability of perovskite NCs.[^14b^, ^17a^, ^36^, ^37^, ^38c^, ^39^] For example, Bodnarchuk et al. demonstrated that treating the surface of aged or purified CsPbBr_3_ NCs with a mixture of didodecyldimethylammonium bromide and PbBr_2_ leads to an improvement in the PLQY up to 90–100 % (Figure 5 a).^[17a]^ The authors proposed that the damaged NC surface is repaired by the ammonium cation occupying the A‐site and the bromide anion occupying the halide vacancies. Surface passivation has resulted in stable colloidal solutions with PLQYs up to 95–98 % even after purifying 3 or 4 times. Another type of ligand family that has received significant attention is zwitterionic ligands (or bidentate ligands), in which the two functional groups with positive and negative charges simultaneously bind to the A‐site and X‐site, respectively.[^17a^, ^21^, ^26g^] Therefore, these ligands bind to perovskite NC surfaces much more strongly because of the chelating effect, and the resulting NCs exhibit long‐term stability and high PLQYs even after washing with different solvents.[^17a^, ^26g^, ^38e^] In contrast to using alkylammonium halides and zwitterionic ligands, Alivisatos and co‐workers proposed that surface treatment with X‐type anionic ligands alone can improve the PLQY of CsPbBr_3_ up to near unity.^[31]^ They proposed that the X‐type ligands passivate the halide vacancies based on hard‐soft acid‐base theory. Their results suggest that halide vacancies are the main nonradiative traps in perovskite NCs (see Section 2 and Figure 3 b). This work has led to debate on the role of A‐site vacancies on the PLQY of perovskite NCs. In addition, a comparison of the PLQY vs. CsPbX_3_ NC concentration, that is, the PLQY vs. density of halide vacancies, for three different halides (Cl^−^, Br^−^, and I^−^) revealed that CsPbI_3_ NCs are tolerant to halide vacancies, whereas the CsPbBr_3_ NCs are moderately intolerant and CsPbCl_3_ NCs are highly intolerant (Figure 3 a). This is consistent with the low PLQYs (1–10 %) of pristine CsPbCl_3_ NCs compared to other halide compositions.^[40]^ On the other hand, because of their defect‐tolerant nature, no improvement is expected in the PLQY of CsPbI_3_ NCs upon surface halide passivation. In fact, most studies related to surface passivation and doping of CsPbI_3_ NCs have been focused on improving their stability rather than the PLQY.^[41]^ This is because of the structural instability of the optically active α‐phase cubic CsPbI_3_ NCs, which generally changes into the nonfluorescent yellow (δ−) phase that is less defect‐tolerant, as discussed previously. However, in some studies, it has been shown that the PLQY of CsPbI_3_ NCs can be improved up to near unity upon surface passivation with bidentate ligands^[38e]^ or by in situ passivation with inorganic salts.^[42]^ It is still unclear whether the ligands passivate the defects or if they improve the phase stability of the CsPbI_3_ NCs. However, despite their defect‐tolerant nature, the origin of the non‐unity PLQY of CsPbI_3_ NCs is quite interesting and needs further investigation.

**Figure 6:**
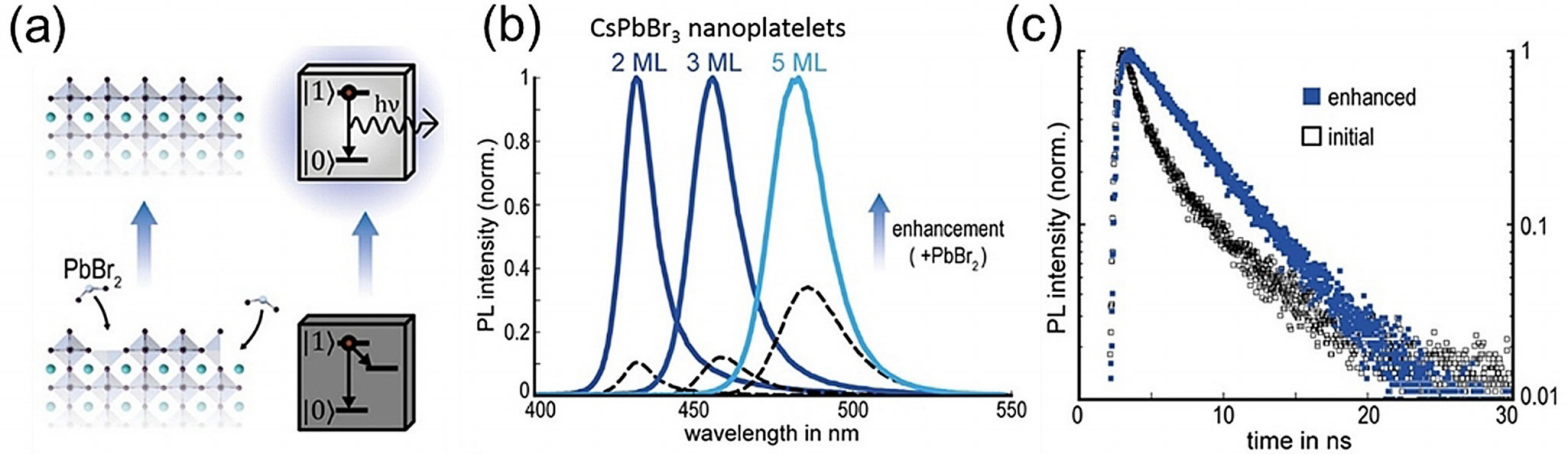
a) Schematic illustration showing the post‐synthetic surface repair of CsPbBr_3_ NPls by chemical treatment with a PbBr_2_ solution to improve their photoluminescence efficiency. b) Photoluminescence spectra of pristine (black dashed lines) and surface‐repaired (normalized, solid lines) colloidal dispersions of CsPbBr_3_ NPls with three different thicknesses. The enhancement clearly increases as the thickness of the NPls decrease, which suggests that the defects are more effective as the surface to volume ratio increases. c) Time‐resolved photoluminescence spectra of a 3‐monolayer NPl dispersion before (open black squares) and after surface repair (filled blue squares). (a,b,c) are reproduced from Ref. [14c] with permission the under Creative Common CC‐BY license. Copyright 2018, American Chemical Society.

Besides organic ligands, metal halides[^14c^, ^38a^, ^38b^, ^43^] and a few other inorganic compounds[^14d^, ^38f^, ^44^] have also been found to be promising for the passivation of halide vacancies of perovskite NCs to improve their PLQYs and long‐term stability. The halide ions of the added metal salts are expected to fill the halide vacancies and thus repair the surface of perovskite NCs. For example, Bohn et al.^[14c]^ found that post‐synthetic treatment of CsPbBr_3_ nanoplatelets (NPls) with a PbBr_2_‐ligand solution leads to a dramatic improvement in the PL intensity (Figure 6). Controlled experiments with different metal halides suggest that both the Pb and Br vacancies are the origin of the lower PLQY of CsPbBr_3_ NPls. The addition of a solution of PbBr_2_ leads to the filling of the Pb and Br vacancies on the surface (Figure 6 a). Interestingly, NPl‐thickness‐dependent surface passivation shows that the PL enhancement factor increases as the NPl thickness decreases (Figure 6 b), which suggests a higher density of halide vacancies with an increase in the surface to volume ratio. In fact, this is consistent with the decrease in the PLQY as the thickness of pristine CsPbBr_3_ NPls decreases.[^14c^, ^45^] Removal of nonradiative traps by treatment with PbBr_2_ is reflected in the increase in the PL lifetime and the shape of the decay profile (Figure 6 c). The PL decay of the treated sample is more monoexponential compared to the PL decay of the pristine NPl sample. Post‐synthetic treatment of CsPbBr_3_ NC films with PbBr_2_ led to a PLQY of near unity.^[38a]^ Similarly, a near unity PLQY of CsPbBr_3_ NPls was achieved by Wu et al. by using in situ passivation with a solution of HBr during synthesis.^[46]^ Compared with all‐inorganic perovskite NCs, in which the surface termination could be either PbX_2_ or CsX and the possible surface defects are Pb, Cs, or halide vacancies,^[17a]^ Bertolotti et al. suggested that the dominant surface termination of NPls are CsX with Cs and halide vacancies in nearly stoichiometric proportion.^[47]^ Therefore, common passivation strategies for NPls reported so far have focused on X‐site vacancy passivation, such as using PbBr_2_ or HBr.[^14c^, ^46^]

Furthermore, treatment with metal halides was found to be very effective for improving the PLQY of weekly emissive CsPbCl_3_ NCs. Since the CsPbCl_3_ NCs are highly intolerant of Cl vacancies, they are weekly emissive. However, treatment with metal chloride leads to the recovery of the damaged PbCl_6_^−^ octahedral coordination and thus significantly improves the PLQY of CsPbCl_3_ NCs. For example, Behera et al. systematically investigated the influence of the treating the surface of CsPbCl_3_ NCs with different metal chlorides, as well as nonmetal chlorides, and they observed an enhancement in the PLQY up to 25–50 %.^[48]^ The origin of the PL enhancement was attributed to the presence of a halide‐rich surface after treatment with metal chlorides. More importantly, the purification process is very sensitive and the PL can be significantly quenched by a phase transformation from CsPbCl_3_ to CsPb_2_Cl_5_. These results further indicate that CsPbCl_3_ NCs are highly intolerant of defects, unlike their Br and I counterparts. Similarly, Ahmed et al. reported that the PLQY of CsPbCl_3_ NCs could be improved up to 60 % by surface treatment with YCl_3_.^[43]^ They proposed that both the Y^3+^ and Cl^−^ ions contribute to the surface passivation in such a way that the Cl^−^ ions bind to under‐coordinated Pb ions and the Pb‐Cl ion pair vacancies will be filled by Y‐Cl. This needs an in‐depth investigation of how the Y^3+^ ions are doped in the CsPbCl_3_ NCs. Similarly, post‐synthetic treatment of CsPbCl_3_ NCs with CdCl_2_ at room temperature leads to an increase in the PLQY from 3 to 98 %.^[38d]^ The origin of the enhancement was attributed to the replacement of Pb^2+^ by Cd^2+^ ions during the cation‐exchange process. The author claimed that CdCl_2_ in the saturated ethanol solution reacted with the CsPbCl_3_ surface to bind with the surface through Cl vacancies. Then, the smaller Cd^2+^ ion partially replaced the larger Pb^2+^ ions and reduced the cuboctahedral voids, thereby leading to less tilting of the octahedral unit and an increase in the tolerance factor.^[49]^ Therefore, with B‐site doping and passivation of the Cl vacancies on the surface by Cl^−^ ions, the CdCl_2_‐treated NCs achieved a PLQY of near unity (96 %).

## 4 Improving the Performance of LEDs through Defect Passivation

**Figure 7:**
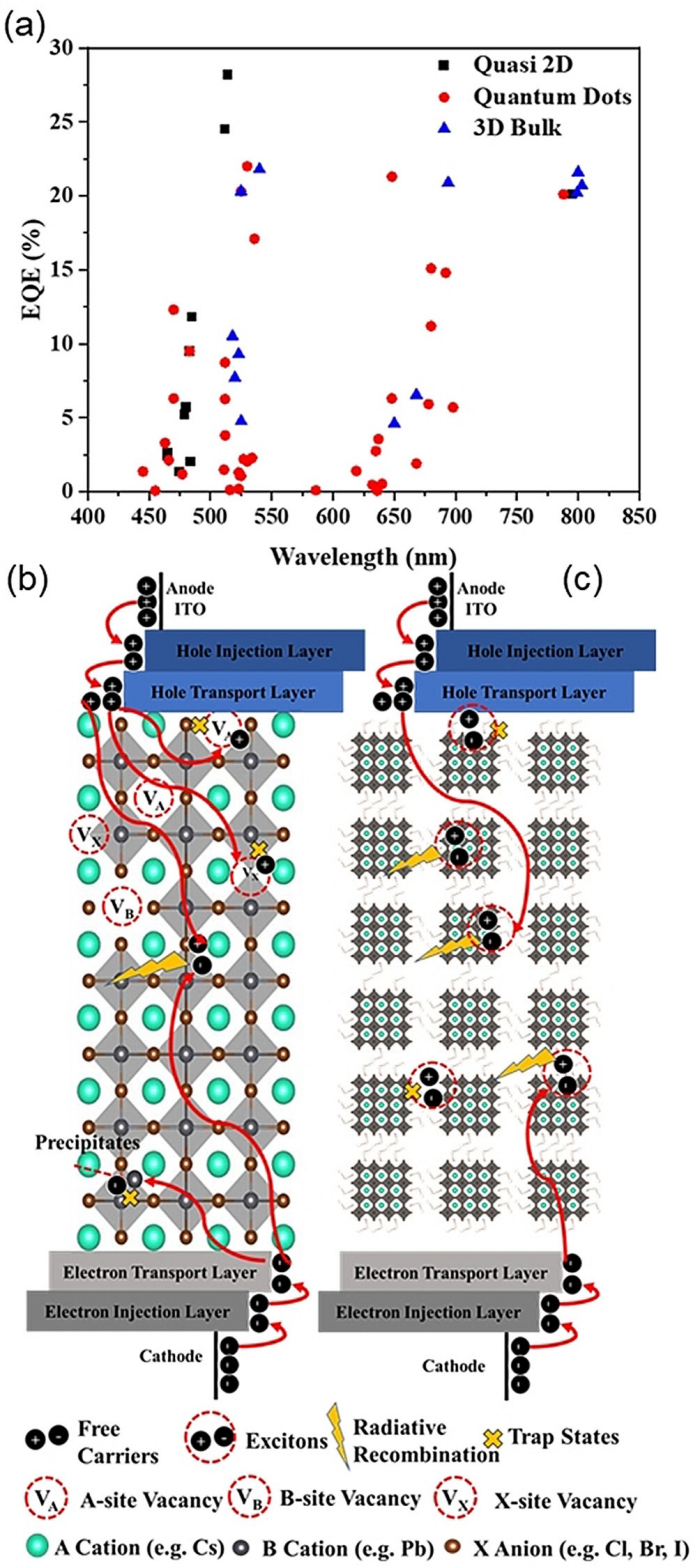
a) External quantum efficiency (%) vs. emission wavelength (nm) of a perovskite light‐emitting diode. b) Radiative recombination and trap states in the dominant free charge‐carrier recombination process in bulk perovskite LEDs. Injected electrons and holes can be trapped in defects before radiatively recombining. c) Dominant exciton recombination process in quantum dot (nanocrystals) perovskite LEDs. Radiative and trap‐assisted monomolecular exciton recombination compete with each other at a low carrier density.

Lead‐halide perovskites have attracted significant attention for applications in LEDs (PeLEDs) owing to the rapid increase in efficiency (from ca. 1 % EQE in 2014 to >20 % EQE in 2020),^[50]^ sharp electroluminescence (EL) bands, color‐tunability over the entire visible‐wavelength range, and versatile, facile processability. In particular, the EL bands exhibited by LHPs are narrower than those of other organic and inorganic materials, which give rise to high color saturation and could lead to displays that emit over a wider color gamut. Although progress has been rapid for green‐, red‐, and near‐infrared‐emitting perovskite LEDs (all with a demonstrated external quantum efficiency (EQE) >20 %, Figure 7 a), blue and pure‐red perovskite LEDs have lagged behind. This limits the possibility of achieving all‐perovskite ultrahigh‐definition displays or using perovskites for solid‐state white lighting. As a result, the past couple of years have witnessed a shift in effort towards blue emitters, and progress has accelerated, with EQEs increasing from 2.12 % in 2018^[51]^ to >10 % today for both blue and sky‐blue emitters (Figure 4 a).^[52]^

From a device point of view, the prerequisite for obtaining an LED with a high external quantum efficiency (EQE) is governed by four factors: 1) charge balance between injected electrons and holes, 2) coupling between electrons and holes, 3) the fraction of all recombination processes that are radiative [PLQY; Eq. 4], and 4) the fraction of photons generated that are emitted out of the device (outcoupling).^[53]^ In this section, we will focus on factor 3, to discuss the methods used to obtain high PLQYs through defect passivation routes. Details of the other factors can be found in Refs. [53, 54].

(4) $$PLQY=\frac{\sum R_{rad}}{\sum R_{rad}+\sum R_{non}}$$

The PLQY is defined as the ratio between the radiative recombination (∑*R*_rad_) and the sum of radiative recombination and nonradiative recombination (∑*R*_non_). In metal halide perovskites, radiative recombination can be from free carriers (bimolecular recombination) or excitons (excitonic recombination). The mechanism for radiative recombination is strongly dependent on the dimensionality of the perovskites and is significantly influenced by the exciton binding energy. In 3D perovskite thin films, the exciton binding energy is below the room‐temperature thermal energy,^[55]^ thereby resulting in excitons readily dissociating into free carriers. The role of excitons in bulk perovskites has been discussed by Marongiu et al., who claimed that the majority of photoexcitations are from free carriers, with only a small fraction of Coulombically bound electron–hole pairs under the typical working regimes of solar cells and LEDs.^[56]^ Therefore, radiative recombination in 3D perovskites is dominated by the recombination of free charge carriers and the PLQY can be calculated by Equation 5, where *a* is the nonradiative trap‐assisted monomolecular recombination coefficient, *b* is the bimolecular recombination coefficient of the radiative free charge carrier, *c* is the nonradiative three‐body Auger recombination coefficient, and *N* is the excess carrier density above background under illumination of the film.[^55^, ^57^] Figure 7 b illustrates the carrier movement in bulk PeLEDs, where the free charge carriers can be easily trapped in material defects such as halide vacancies, A‐site vacancies, anti‐sites, and Pb precipitate clusters,^[2c]^ because the bimolecular recombination rates are usually slow in bulk perovskites.^[58]^ However, the actual chemical equilibrium between free carriers and excitons in 3D PeLEDs is still under debate. Recent experiments showed that emission spectra were well‐reproduced at the energy of the exciton absorption, where the contribution from band‐to‐band transitions is negligible, thus implying that luminescence is mainly due to the recombination of excitons. Despite, being outnumbered by free carriers in bulk perovskite films, excitons emit most of the light, as their faster radiative decay rate more than compensates for their lower density.[^56^, ^59^] Hence, more studies are necessary to fully understand the generation of free carriers and excitons as well as the radiative recombination process in PeLEDs.

(5) $$PLQY=\frac{bN}{a+bN+cN^{2}}$$

In contrast, radiative recombination in quantum‐confined perovskites is dominated by excitonic monomolecular recombination due to the relatively high exciton binding energy (usually hundreds of millielectronvolts, ca. 190–500 meV).^[60]^ Therefore, the PLQYs for perovskite quantum dots or nanocrystal systems are strongly dependent on the competition between the first‐order radiative excitonic recombination and the first‐order nonradiative exciton trapping, as shown in Equation 6, where *k*_ex,_ *k*_trap_, and *k*_Aug_ are the radiative monomolecular exciton recombination coefficient, nonradiative monomolecular exciton trapping coefficient, and nonradiative Auger recombination coefficient, respectively. Figure 7 c demonstrates the exciton recombination and trapping process in perovskite nanocrystal LEDs. The presence of surface ligands significantly reduces the number of perovskite defects compared with bulk 3D perovskites; as a result, perovskite nanocrystals can achieve PLQYs near unity. As discussed in the previous section, surface charged defects arising from aging or washing can act as exciton trapping centers and hinder the device performance. Another exciton‐loss mechanism in quantum dot systems is through exciton–ligand interactions. Kawano et al. confirmed that the Wannier and Frenkel exciton energies exhibit near‐resonance, which leads to selective emission quenching in the organic ligands (naphthylmethyl moieties). They explained the selective luminescence quenching by an enhancement in the oscillator strength of near‐resonant transition energies between Wannier and Frenkel excitons. This provides potential evidence that ligands can also act like an exciton trapping center to reduce the desirable radiative recombination.^[61]^ The exciton–ligand interactions have also been studied in a traditional PbS quantum dot system. Papagiorgis et al. studied the exciton charge transfer dynamics between PbS quantum dots and small metal chalcogenide ligands (e.g. K_4_GeS_4_). The authors suggest that the excitons can be trapped by the inter‐dot ligands, which quench the photoluminescence from the core‐level excitons.^[62]^ Additionally, Rossi et al. reported that Förster resonance energy transfer occurs between CsPbBr_3_ nanocrystals and ligands based on perylene diimide, which causes emission from the ligands.^[63]^ Based on previous studies of traditional quantum dot systems, such as PbS and CdS,^[64]^ we expected to have ligand–exciton interactions or trapping phenomena. However, the ligands need to be strongly coupled to the electronic structure of the quantum dots (QDs), which can provide additional energy states to trap the excitons.^[64c]^ Additionally, the position of the singlet or triplet states of the organic ligands relative to the conduction band minimum of the QDs are expected to be in the resonance of the incident light for sufficient energy transfer from the QDs to ligand shells.[^64a^, ^64b^] However, detailed studies on perovskite NC systems are still required to understand ligand–exciton coupling and improve exciton transfer between NCs for LED and solar cell applications.

(6) $$PLQY=\frac{k_{ex}}{\left(k_{ex}+k_{trap}\right)+k_{Aug}N}$$

In summary, bulk perovskite thin films have advantages for making solar cells, as carriers can be easily extracted (low exciton binding energy and high mobility). Currently, the record PCE of a perovskite solar cell (25.5 %)^[9]^ was achieved with bulk perovskite thin films, whilst the highest PCE of a perovskite nanocrystal solar cell is 16.6 %.^[65]^ The primary reason for this considerable gap in the PCE is the capping agents of the perovskite nanocrystals, that is, oleic acid and oleylamine. The insulating nature of these ligands limits charge separation and transport, thereby leading to lower carrier extraction and lower PCEs in solar cells. In comparison, perovskite nanocrystals have advantages for making LEDs as they can achieve unity PLQY and higher exciton binding energy through the presence of surface ligands. However, as a result of the insulating nature of long‐chain ligands, the efficiency of nanocrystal PeLEDs can also be low even they can achieve high PLQYs (Figure 7 a). Therefore, ligand engineering and defect management is key for both bulk and nanocrystal PeLEDs to balance the carrier injection efficiency and PLQYs (related to surface defects).

**Figure 8:**
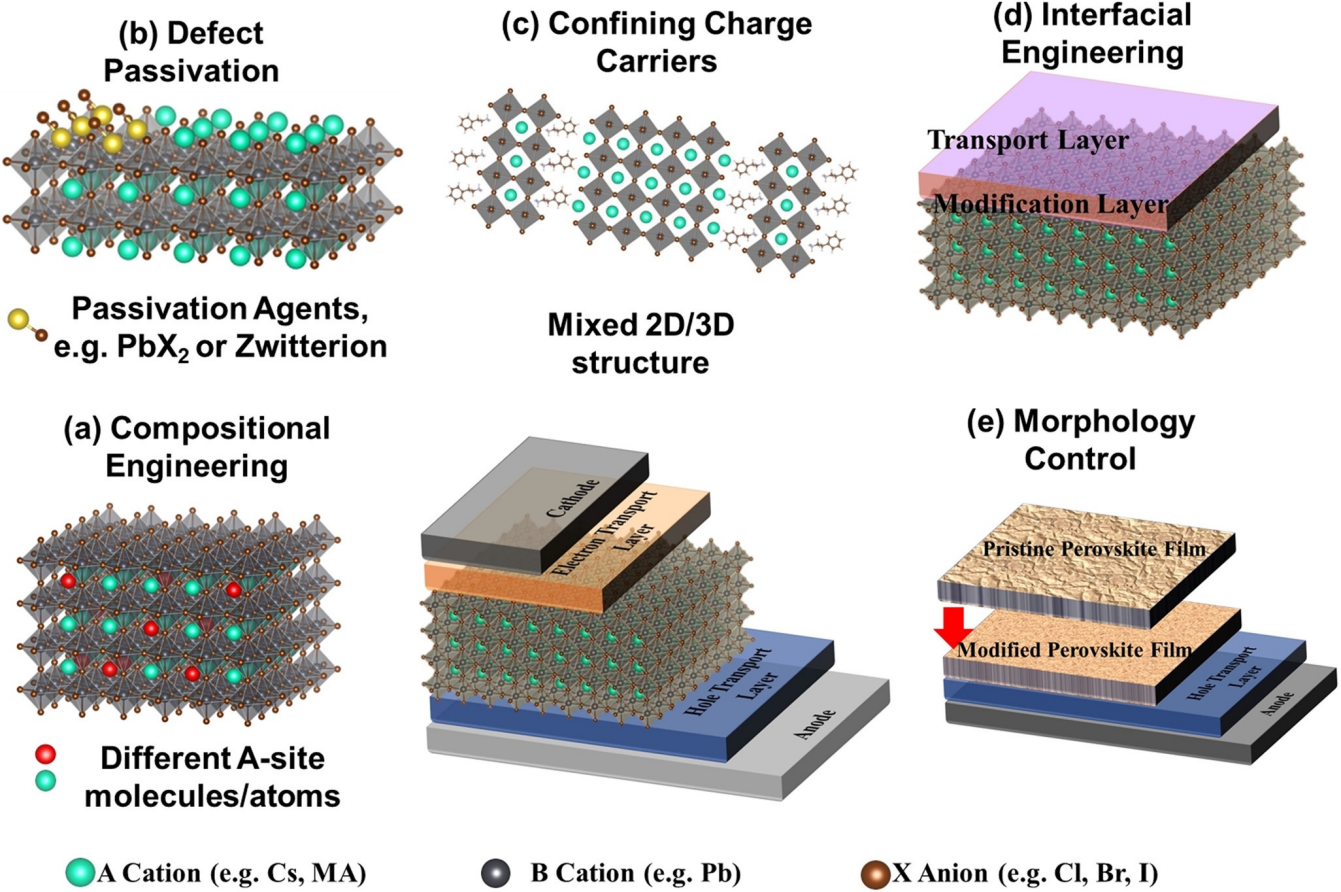
a) Adopting compositional engineering to reduce the formation of intrinsic material point defects. b) Passivating the intrinsic material point defects and surface defects through ligands as well as organic and inorganic salts. c) Maximizing the radiative recombination rates so that less nonradiative recombination occurs by confining the charge carriers. d) Passivating interfacial defects through interfacial engineering, such as by adding a modification layer. e) Morphology control to improve the film morphology and minimize grain boundary defects.

The general principle for designing high‐performance PeLEDs is to maximize the free charge carriers or exciton radiative recombination and minimize nonradiative recombination through the Auger process and traps.^[66]^ There have been many detailed reviews about modifying the charge‐transport layer to improve the charge balance and reduce the Auger process,^[67]^ including improving hole injection by adding an extra polymer modification layer to achieve better band alignment^[68]^ as well as doping hole transport material to increase hole mobility^[69]^ and decrease charge leakage.^[70]^ Another challenge for PeLEDs and perovskite solar cells is operational instability. The origin of this instability mainly arises from ion migration in the perovskite layers.^[71]^ Ion migration is intrinsically a defect migration process.^[72]^ The drift‐diffusion model is usually used to model the net current in PeLEDs and solar cells,[^73^, ^74^] in which the drift current is induced by an external electrical bias and the diffusion current is governed by a carrier concentration gradient. Under an applied electrical bias, the ions (halide ions or A‐site cations) can migrate via defects, thereby causing material degradation which is irreversibly detrimental to device stability.^[72a]^ Methods such as making diffusion‐driven charge‐transport LEDs^[75]^ and effective diffusion engineering of the charge injection layers^[76]^ would be future directions to suppress ion migration in planar LEDs. In this Review, we will only focus on improving PeLED performance by mitigating the effects of defects to improve device efficiency and stability (Figure 8).

**Figure 9:**
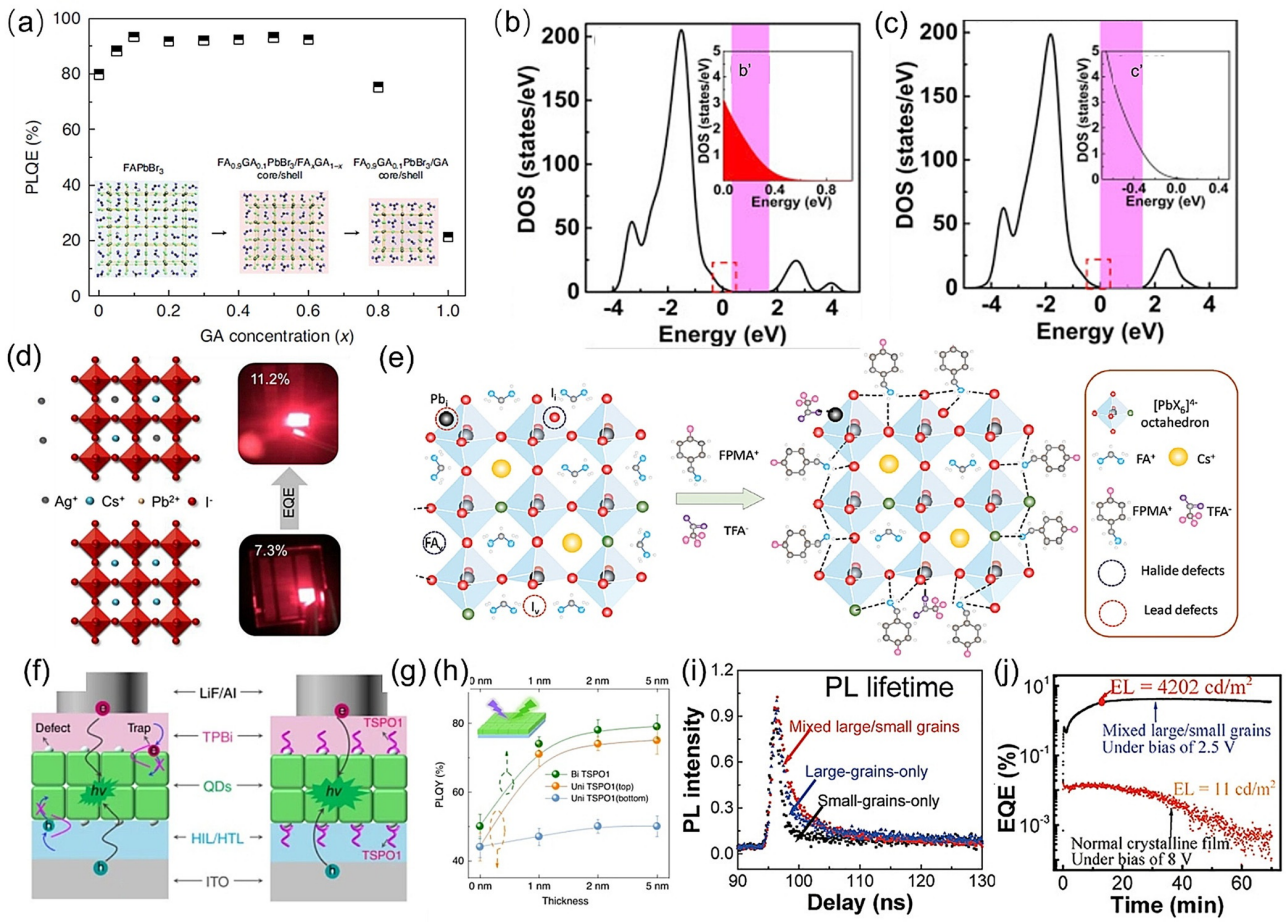
a) Photoluminescence quantum efficiency vs. GA doping concentration and schematic crystal structures (inset) of FA_1−*x*_GA_*x*_PbBr_3_ PNCs. Reproduced from Ref. [77] with permission. Copyright 2021, the authors, under exclusive licence to Springer Nature Limited b,c) The insets b′ and c′ show larger versions of the highlighted regions (red box). The band gap position is illustrated in purple. Reproduced from Ref. [78a] with permission. Copyright 2020, Elsevier Ltd. All rights reserved. d) Illustration of the increase in the EQE upon Ag doping. Reproduced from Ref. [79] with permission. Copyright 2018, American Chemical Society. e) Schematic illustration of defect passivation at perovskite grain boundaries by FPMATFA. Reproduced from Ref. [52h] with permission. Copyright 2020, WILEY‐VCH Verlag GmbH & Co. KGaA, Weinheim f,g) Structure of the QLED based on QD films without passivation (f) and with passivation (g). It is shown that TSPO1 could passivate defects on the surface of QD films, the defects may trap carriers (e.g. holes, electrons), decrease exciton recombination, and hence degrade the device performances. h) PLQY of QD films without and with TSPO1 on the bottom, on the top, and on both sides of a QD film. Error bars represent the standard deviation of experimental data for three measurements. Reproduced from Ref. [92] with permission under the Creative Common CC‐BY license.Copyright 2020, the authors. i) PL lifetimes for films with mixed large/small grains, small‐grains only, and large‐grains only. j) EQE as a function of time under a constant bias for perovskite LEDs with mixed large/small grains (black curve) and a normal perovskite film prepared by the vapor‐assisted two‐step method (red curve). Reproduced from Ref. [97] with permission under the Creative Common CC‐BY license. Copyright 2019, the authors.

The first common method to suppress nonradiative recombination is through compositional engineering, including A‐site,^[77]^ B‐site,^[78]^ and X‐site^[52g]^ tuning to improve the PLQY. The mechanism consists of the following: 1) Increasing the defect formation energy and reducing the intrinsic defects of the materials by extra interactions between the dopants and perovskite lattice.[^77^, ^78^] For example, Kim et al. doped guanidinium cations (GA^+^) into the A‐site of FAPbBr_3_ NCs; the amine group of the GA cation can interact with lattice halides through hydrogen bonding between the amine group and the Pb‐halide framework which improves the material stability and reduces defect formation.^[77]^ As a result, the PLQE increases from 79.7 % (pristine FAPbBr_3_ NCs) to 93.3 % (FA_0.9_GA_0.1_PbBr_3_ NCs; Figure 9 a). Similarly, Zhang et al. reported that by B‐site doping of CsPb(Br_*x*_Cl_1−*x*_)_3_ with La^3+^ ions, the density of state in the conduction band is increased, which inhibits the formation of defects at mid‐band gap states (Figure 9 b,c).^[78a]^ As a result, the radiative recombination rate increased from 0.036 ns^−1^ (0 % La^3+^) to 0.069 ns^−1^ (5.43 % La^3+^) with an increase in the PLQY from 3.47 % to 84.3 % and an increase in the EQE from less than 0.5 % to 3.25 % at 489 nm. 2) The surplus of the doping agent can passivate surface defects and dangling bonds to reduce nonradiative recombination sites.[^77^, ^79^] Kim et al. suggested that the excess of the GA^+^ dopant can modulate the surface of the NCs and passivate the surface defects to achieve a high‐performance green LED with a current efficiency of 108 cd A^−1^ and an EQE=23.4 %. Similarly, Lu et al. doped Ag^+^ ions into CsPbI_3_ NCs. The addition of the Ag^+^ ions is believed to be beneficial for surface passivation as well as doping (Figure 9 c). The diffusion of Ag ions into the perovskite lattice stabilizes the CsPbI_3_ NCs, while the excess Ag ions on the NC surface reacts with iodide to form AgI, which also partially stabilizes the NCs from moisture and irradiation. The simultaneous silver doping and passivation lead to an increase in the device performance from 7.3 % to 11.2 %.^[79]^ 3) Doping elements with a smaller atomic radius could increase the exciton binding energy and lead to high radiative recombination rates, as the exciton lifetime is inversely proportional to the binding energy.[^78a^, ^80^] Furthermore, for CsPbI_3_ systems, partially replacing the Pb^2+^ by smaller Mn^2+^,^[81]^ Sr^2+^,^[82]^ or Zn^2+[83]^ ions leads to a reduction in the strain imposed by the larger iodides. This occurs by increasing the cohesive energy among the octahedral units and, as a result, it restricts the transition from the facile photo‐active alpha‐phase to the photo‐inactive delta‐phase and thus provides higher thermal stability.^[14b]^

Various types of passivation agents have been extensively discussed in Section 3, including organic ligands (such as Lewis acids/bases,[^38e^, ^52i^] amine‐based passivation materials such as branched polyethyleneimine (PEI) and ethylenediamine (EDA)^[84]^), inorganic salts (such as lead halides[^14c^, ^68a^] and potassium halides,^[85]^ potassium salts (KPF_6_)),^[86]^ and organic salts.[^52g^, ^86^, ^87^] The passivation of intrinsic material defects can be achieved by loading the passivation agents into the precursors prior to film deposition or colloidal synthesis[^85^, ^88^] or during post‐treatment of the crystallized perovskite films^[89]^ or colloidal solution.[^52g^, ^52l^] Furthermore, treatments carried out during deposition of the film can benefit the device performance. Recently, Karlsson et al. developed a vapor‐assisted crystallization method to reduce the density of surface defects.^[89]^ Directly after spin‐coating, the as‐deposited perovskite thin films were placed in a petri dish under an *N*,*N*‐dimethylformamide (DMF) atmosphere. The presence of DMF vapor allows redistribution of the surface halides, which leads to a more homogenized surface halide composition with a lower defect density, enabling an increase in the film PLQY from 3 % (control) to 12 % (VAC‐treated), as well as an increase in the PL lifetime. By doing so, they achieved a more spectrally stable blue LED with the EQE increasing from 0.6 % (without VAC treatment) to an average of 3.8 % (with VAC treatment).^[89]^ Another interesting concept for defect passivation is to design the passivation agents so that they are suitable for passivating multiple types of defects. Fang et al. reported a dual passivation of perovskite defects using a bifunctional molecule, 4‐fluorophenylmethylammonium trifluoroacetate (FPMATFA). The TFA anions and FPMA cations can bond with undercoordinated lead and halide ions, respectively (Figure 9 e), thereby resulting in dual passivation of both lead and halide defects. This dual passivation method results in an efficient LED device with 20.9 % EQE at *λ*=694 nm.^[52h]^

The bimolecular recombination rate for bulk perovskite LEDs is low, which leads to a low PLQY, and the electrons and holes can be easily disassociated and trapped in defects. Therefore, another approach is to improve the PLQY of the intrinsic material by increasing confinement effects through heterostructures, such as by constructing a mixed 2D/3D (quasi‐2D) structure[^52c^, ^90^] or through perovskite‐polymer heterostructures,[^52k^, ^91^] which could prolong the carrier lifetime and improve the structural stability. The 2D/3D passivation strategy is discussed in detail Section 5. Furthermore, the presence of polymer, such as poly(2‐ethyl‐2‐oxazoline (PEOXA), can regulate the crystallization process to further reduce boundary defects.^[91b]^ Cai et al. showed that the polymer matrix provides excess nucleation sites during the NC recrystallization process, which leads to more uniform distributions of NCs in the films, thereby resulting in a higher PLQY and EQE (from 1.4 % with 0 wt % polymer to 6.55 % with 45 wt % polymer) in thin films of the composite.^[91b]^

Interfacial engineering is also critical to achieve high‐performance PeLEDs. It is suggested that defects can be most easily generated on the surface of as‐deposited perovskites as there is a significant number of dangling bonds at the exposed interfaces which would quench photoluminescence.^[67b]^ Furthermore, the wettability of the surface onto which the perovskite is grown is critical for the crystallization of the solution‐ deposited perovskites, as it directly influences the concentration of GB defects in the films.[^52f^, ^67b^] As illustrated in Figures 7 b and 9 f, defects between transport layers and emissive layers can trap carriers which reduce radiative recombination. Several reviews have been published that focus on interfacial layer engineering for solar cells and LEDs, such as improving the charge injection and charge balance, suppressing interfacial defects, and exciton quenching.[^2b^, ^67b^] In contrast to traditional one‐sided interfacial engineering, Xu et al. reported a bilateral interfacial passivation strategy by evaporating organic ligand molecules (diphenylphosphine oxide‐4‐(triphenylsilyl)phenyl (TSPO1)) on both the top and bottom of the perovskite emitting layer (Figure 9 g). By doing so, the film PLQY was enhanced (Figure 9 h). The interactions between uncoordinated Pb bonds and P=O bonds from TSPO1 can significantly suppress interfacial nonradiative recombination and improve the EQE from 7.7 % (nonpassivated) to 13.5 % (passivated only on the top) and 18.7 % (passivated on both sides).^[92]^ Therefore, it would be a novel approach to incorporate interfacial passivation on both sides of perovskites in the design of future devices. Apart from adding a film, mechanically removing the defective surface could be another interesting approach to try to modify interfacial defects in PeLEDs. Chen et al. removed the defective MAPbI_3_ perovskite surface layers by using adhesive tape to mechanically peel off the top surface. By doing so, the PL intensity of the film increased, which indicates suppression of surface nonradiative recombination.^[93]^ This could be a useful approach for PeLED applications.

Surface morphology and grain engineering are also used for improving PLQYs to achieve more efficient PeLEDs. The film morphology of perovskites is strongly dependent on the deposition conditions, including solution concentration, solvents, spin conditions, annealing temperature, the wettability of the substrates, and how supersaturation is achieved (e.g. antisolvent dropping during spin‐coating or gas‐phase quenching).^[94]^ Yu et al. conducted a detailed review on the effect of perovskite film morphology on LED device performance.^[94]^ Both 3D and NC perovskites are strongly dependent on the surface ligands used.^[95]^ Beyond surface passivation, appropriate ligand engineering is needed to obtain uniform and pinhole‐free films.^[96]^ Qin et al. demonstrated that the grain size of the perovskite films plays a role in carrier lifetimes and device efficiency.^[97]^ Attaching high‐band‐gap small grains to the surfaces of low‐band‐gap large grains can form interfacial potential wells that can spatially confine GB defects and mobile ions to enable self‐passivation during device operation. In addition, the small nanometer‐sized grains can effectively confine injected electrons and holes and then efficiently cascade the injected carriers from high‐band‐gap small grains to low‐band‐gap large grains that function as light‐emitting centers. Furthermore, attaching small grains to the surfaces of large grains leads to high‐quality films for device fabrication. With this design, self‐passivation can be realized when mobile ions are electrically driven to GBs to neutralize the charged defects, and as a result the carrier lifetime and device EQE is increased (Figure 9 i,j).^[97]^

## 5 Improvement of the Performance of Solar Cells by Defect Passivation

Defect passivation strategies are also extensively studied to improve the power conversion efficiency of perovskite solar cells (PSCs). Many reviews have summarized the influence of the defects on charge‐carrier recombination and transport, ion migration, and the structural stability of solar cell devices, as well as summarizing methods to passivate defects and improve device performance.[^2b^, ^98^, ^99^] In this section, we will focus on passivation methods for 2D/3D perovskites.

### 5.1 2D/3D Perovskite Passivation

**Figure 10:**
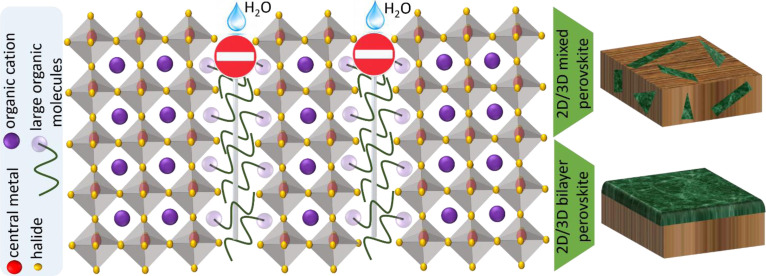
Schematic illustration of a 2D/3D perovskite structure: passivation, stability, and forming the mixed and bilayer structures.

Typically, as a result of the polycrystalline structure and ionic nature of organic–inorganic perovskites, structural defects in the bulk, at GBs, and at the surface need to be considered and passivated to suppress the nonradiative recombination in perovskite films.^[100]^ For example, the evaporation or migration of some volatile components such as methylammonium (MA) or to a lesser extend iodine (I) can lead to the formation of cation or halide vacancies in three‐dimensional (3D) perovskites.^[101]^ So far, numerous organic^[102]^ and inorganic^[103]^ materials have been used for passivating the different types of defects. Large organic molecules (LOMs) with appropriate functional groups can not only passivate the defect states but also stabilize the 3D perovskite structure by forming a 2D perovskite at the GBs or the surface of the 3D films, and this is termed a 2D/3D structure.^[104]^ Therefore, in addition to suppressing the interfacial charge‐carrier recombination, the moisture‐entry pathways could be suppressed because of the hydrophobic nature of the LOMs. This results in improving the efficiency and the stability of perovskite solar cells (PSCs) simultaneously (Figure 10). However, various 2D/3D structures can be formed by applying different treatment approaches to the perovskite films. For example, adding the LOMs to the perovskite precursor solution yields a 2D/3D mixed structure, while post‐treatment of the perovskite films with the LOMs tends to form a 2D/3D bilayer structure (Figure 10). The passivation of perovskite films with LOMs by creating different 2D/3D structures will be discussed in the following section.

### 5.2 2D/3D Mixed Perovskites

**Figure 11:**
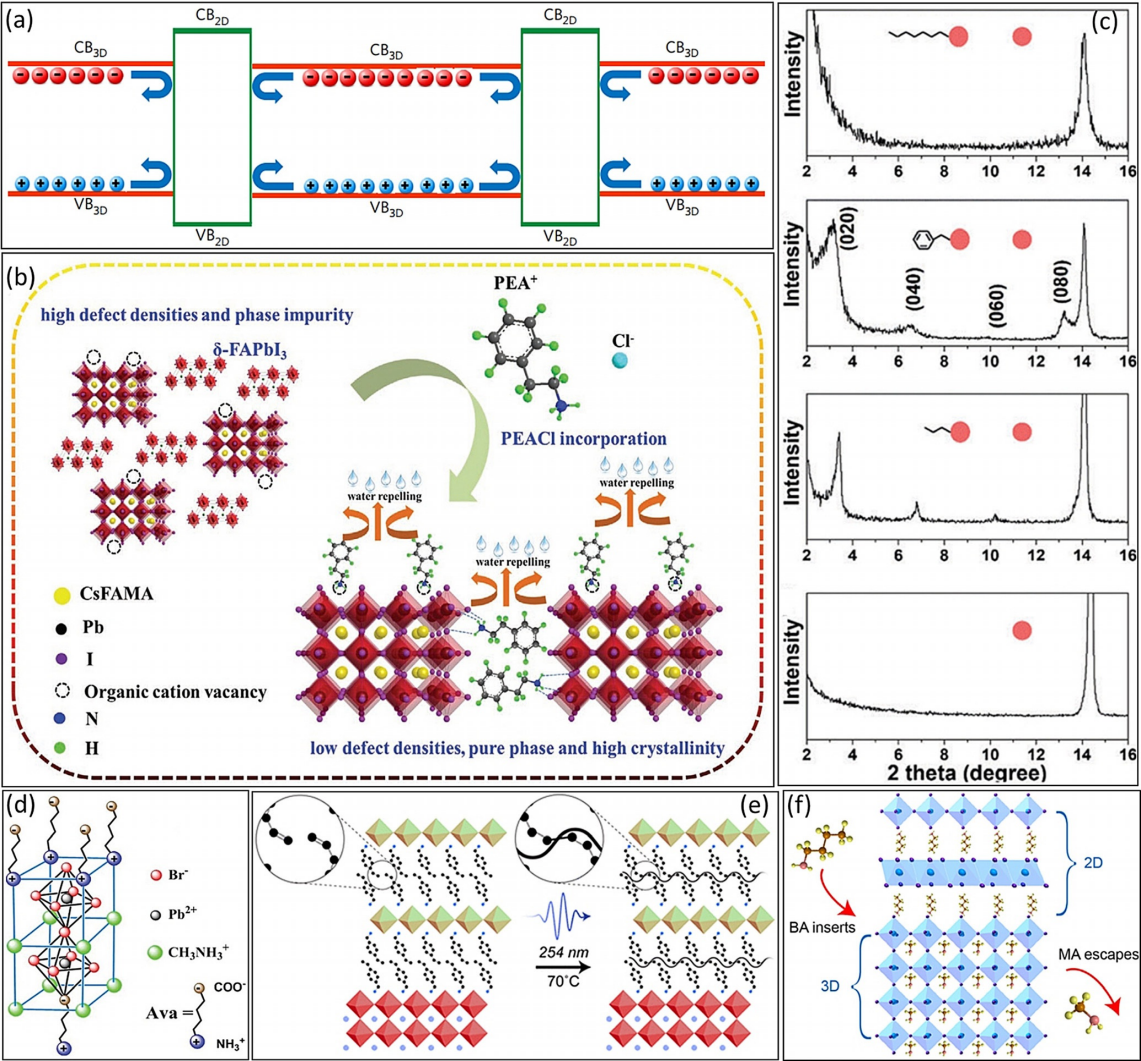
a) The proposed electronic interface in a 2D/3D mixed structure. Reproduced from Ref. [106a] with permission. Copyright 2017, Springer Nature. b) The proposed mechanism for the passivation of 3D perovskite with PEA^+^ ions. Reproduced from Ref. [109b] with permission. Copyright 2020, John Wiley and Sons. c) The XRD pattern of perovskite films without and with PEA^+^, BA^+^, and OA^+^ additives. Reproduced from Ref. [110] with permission. Copyright 2018, the Royal Society of Chemistry. d) Schematic chemical structure of a 5‐AVA cross‐linked perovskite structure. Reproduced from Ref. [111] with permission. Copyright 2017, John Wiley and Sons. e) Schematic illustration of the formation of a cross‐linked 2D/3D bilayer perovskite. Reproduced from Ref. [112] with permission. Copyright 2019, American Chemical Society. f) The mechanism of forming the 2D/3D bilayer structure by treatment with BA^+^ vapor and time‐resolved PL spectra of 3D and 2D/3D bilayer perovskites. Reproduced from Ref. [113] with permission. Copyright 2019, American Chemical Society.

LOMs have been used for creating a pure 2D Ruddlesden–Popper (RP) perovskite L_2_A_*n*−1_B_*n*_X_3*n*+1_ structure, where L is a bulky OM spacer, A is a monovalent cation such as Cs^+^, MA^+^, or formamidinium (FA^+^), B is a central metal ion (Pb^2+^ or Sn^2+^), X is a halide (I^−^, Cl^−^, or Br^−^), and *n* (1, 2, 3, 4, …. ∞) is the number of inorganic perovskite layers within each quantum well.^[105]^ Although 2D PSCs have undergone tremendous development in terms of their stability in the last few years, they still suffer from poor charge transport because of the insulating nature of the inserted LOM spacers in their structures, which result in lower efficiencies achieved compared to 3D PSCs.^[11]^ To answer this challenge, combining 2D and 3D perovskites has been introduced as an efficient approach to accomplish both high stability (from the 2D structure) and high efficiency (from the 3D structure) simultaneously.^[106]^ The 2D/3D mixed structure can be formed by adding the LOMs directly to the perovskite precursor solution. Nevertheless, various LOMs with different functional groups can be used to passivate different types of defects in perovskite films. The cationic LOMs with ammonium (NH_3_^+^) functional groups have been used widely for defect passivation and to form a 2D/3D mixed structure.^[107]^ The butylammonium (BA^+^)[^106a^, ^107^, ^108^] and phenylethylammonium (PEA^+^)^[109]^ ions are the two most widely used LOMs in this regard. The Snaith group^[106a]^ demonstrated that adding BA^+^ to a perovskite precursor delivers 2D platelets between highly orientated 3D perovskite grains after crystallization, thereby leading to defect passivation and an improvement in moisture stability. They elucidated that the 2D platelets at the GBs of the 2D/3D perovskite provide a clean electronic interface to reflect the charge carriers into 3D grains (Figure 11 a), thereby improving the carrier lifetimes through suppressed charge trapping and recombination. Additionally, the 2D/3D perovskites maintained 80 % of their initial PCE after 1000 h in air, and close to 4000 h when encapsulated.

Ho‐Baillie and co‐workers^[109a]^ showed that adding phenethylammonium ions (C_6_H_5_CH_2_CH_2_NH_3_^+^, PEA^+^) to the perovskite precursor results in the formation of a quasi‐2D structure at the 3D perovskite GBs, thereby leading to in an improved carrier lifetime and increased V_OC_ from 0.99 to 1.10 V for the (FAPbI_3_)_0.85_(MAPbBr_3_)_0.15_ perovskite. Indeed, the Huang group^[109b]^ proposed a mechanism for the passivation of 3D perovskites with PEA^+^ ions. As shown in Figure 11 b, PEA^+^ prefers to either occupy the cation vacancies at the perovskite grains by ionic bonding or interact with undercoordinated I^−^ ions through hydrogen bonding. Moreover, the bulky hydrophobic aromatic ring of the attached PEA^+^ ion at the perovskite surface or GBs can improve the moisture tolerance (Figure 11 b).

The length of the alkyl chain in LOMs is critical to forming a 2D/3D mixed structure. The Seok group compared the crystallization of the 2D/3D structure with BA^+^ and PEA^+^ ions, as the most common 2D agent additives, and with octylammonium (OA^+^) ions having a larger organic chain.^[110]^ In contrast to the case with BA^+^ and PEA^+^ ions, no XRD peak corresponding to the 2D phase was observed for the OA‐incorporated perovskite film (Figure 11 c). The authors proposed that the OA^+^ cations form strong van der Waals forces with the long alkyl chains, and a hydrogen bond forms between the ammonium and under‐coordinated iodide ions, that is, cation vacancies are passivated without creating a 2D/3D structure. Thus, the OA^+^ cations only encapsulated individual 3D perovskite crystal domains with a highly preferential orientation, thereby suppressing the nonradiative charge carrier recombination. As a result, the V_OC_ and FF values of the OA‐passivated PSC were increased from 1.06 V and 79.0 % to 1.11 V and 81.5 %, respectively, thus achieving a higher PCE of 20.6 % than the control PSC (18.4 %). Similarly, very recently, the Bakr group^[114]^ observed that the 2D phase was not formed in the perovskite film with additives having very long‐chain cations, such as OA^+^ and oleylamine (OAm^+^).

On the other hand, passivating the anion vacancies in 3D perovskites, another defect source that effects nonradiative recombination, is very important. One interesting strategy is the use of anionic additives with cationic LOMs. In this regard, Kim et al.^[115]^ demonstrated that the coupling of PEA^+^ cations and SCN^−^ anions can passivate negatively and positively charged defects, that is, cation and halide vacancies, respectively. In addition, the GBs were passivated efficiently by the quasi‐2D perovskite (PEA)_2_Pb(I_4−2*x*_SCN_2*x*_), with its larger band gaps compared to 3D perovskites. As a result, the average PSC performance increases from 16.3 to 18.7 % for the passivated wide‐band‐gap (1.68 eV) PSCs with <4 % degradation after storage for over 4000 h under N_2_. Nevertheless, the passivated perovskite/CIGS 4‐terminal tandem solar cell showed a high PCE of 25.9 %. Another strategy would be to use zwitterion LOMs, which have both cationic and anionic functional groups in their molecular structures. 5‐Aminovaleric acid cations (HOOC(CH_2_)_4_NH_3_^+^, 5‐AVA^+^ hereafter) is one of the most frequently used zwitterions for the passivation of perovskite films through the formation of 2D/3D mixed perovskite structures. Zhang et al.^[111]^ proposed that in the 5‐AVA(MAPbBr_3_)_2_ perovskite structure, the NH_3_^+^ end groups of the 5‐AVA^+^ occupy the MA^+^ positions and the COO^−^ group occupies the Br^−^ sites, thus passivating the cation and anion vacancies and forming cross‐linked 2D/3D perovskites (Figure 11 d). Moreover, the Nazeeruddin group^[106b]^ demonstrated that adding 5‐AVA^+^ cations to the perovskite precursor can form an ultrastable 2D/3D perovskite film, thereby resulting in PSCs that are stable for one year under one sun AM 1.5 G conditions at a temperature of 55 °C.

### 5.3 2D/3D Bilayer Perovskites

Apart from the 2D/3D mixed perovskites, depositing a solution of LOMs on the top of 3D perovskite films can passivate the defects on the surface and form a 2D overlayer, that is, a 2D/3D bilayer. Nevertheless, in contrast to 2D/3D mixed structures, this structure could create a flat or ordered band alignment distribution between the 3D and 2D perovskite phases, which is more favorable for charge transfer and for suppressing unfavorable charge recombination. Similar to the 2D/3D mixed structures, many ammonium‐based cationic LOMs have so far been used for the passivation of cation vacancies on the surface of perovskite films. Docampo and co‐workers^[116]^ developed a facile solution‐based infiltration of BA^+^ and PEA^+^ cations to create a layered 2D/3D perovskite film. They demonstrated that, in this bilayer structure, the 2D layer acts as a selective hole extraction layer between the 3D perovskite and spiro‐OMeTAD, thereby leading to reduced recombination losses and an enhanced V_OC_ value from 0.99 to 1.08 V for both the BA^+^‐ and PEA^+^‐passivated MAPbI_3_ PSCs and with enhanced moisture stability. Many research groups tried subsequently to improve the photovoltaic parameters of PSCs by the surface passivation of perovskite films with these two LOMs.[^108a^, ^117^]

Modification of LOM structures has been introduced as an efficient approach to improve their passivation effects. For example, the Sargent group^[112]^ designed 4‐vinylbenzylammonium (VBA) molecules, with an additional vinyl group compared to the PEA^+^ cations, which are capable of forming a cross‐linked 2D structure by a photochemical reaction under ultraviolet light (Figure 11 e). The cross‐linked 2D/3D perovskite structure showed lower nonradiative recombination compared to the pure 3D structure, with a significant improvement in the V_OC_ value up to 1.20 V and a PCE of up to 20.4 % with negligible J‐V hysteresis when using (MAPbBr_3_)_0.15_(FAPbI_3_)_0.85_ (containing 5 % Cs). Moreover, the cross‐linked 2D/3D PSC retained a PCE of 90 % of its initial efficiency after long‐term stability measurements for 2300 h in air.

Zwitterions with their ability to react with both cation and anion vacancies can be used for the surface passivation of perovskite films and formation of a 2D/3D bilayer structure. For example, the Park group^[118]^ deposited 5‐AVA^+^ cations on the perovskite surface to form an ultrathin 2D (5‐AVA)_2_PbI_4_ passivation layer at the interface of the 3D perovskite and hole transporter layer. The PCE of the passivated PSC was improved substantially from 13.72 to 16.75 %, mainly due to enhanced V_OC_ and FF values, which was attributed to enhanced carrier lifetimes by effective passivation of the defect and trap states on the surface of the 3D perovskite.

In solution‐processed 2D/3D bilayer passivation, however, protic polar solvents (e.g. isopropyl alcohol, IPA) are frequently used to prepare the solution of LOMs. Recently, Bawendi and co‐workers^[13]^ demonstrated that the solvent of the LOM solution, that is, IPA, can dissolve some of the perovskite components, such as FA^+^ ions, thereby degrading the underlying perovskite film. On the other hand, it must be considered that controlling the thicknesses and uniformity of the 2D overlayer formed through solution‐processed approaches is very challenging. To answer this, the Chen group^[113]^ recently exposed the 3D perovskite film to BA^+^ vapor (BAV) to form a uniform 2D overlayer with a tuneable thickness (Figure 11 f). The average PCEs of BAV‐treated PSCs were increased by 10 %, which was ascribed mainly to the higher V_OC_ and FF values compared to control devices. Characterization by photoluminescence spectroscopy showed that the lifetime of the charge carriers was increased from 129.0 ns for the 3D film to 165.5 ns for the 2D/3D film, thus confirming the defect‐passivation effect and suppression of nonradiative charge recombination.

The most frequently used LOMs for passivating perovskite films by creating 2D/3D mixed and bilayer perovskites are summarized in Table 1. It should be noted that besides perovskite solar cells, the 2D/3D structures have shown great potential to improve the efficiency and stability of perovskite LEDs by tuning the radiative recombination coefficient without significant charge‐carrier losses.[^119^, ^120^] For example, Zhang et al. introduced a facile self‐organized and controllable 2D/3D mixed perovskite to improve the performance of blue perovskite LEDs.^[121]^ They demonstrated that the 2D/3D structure increases the electron and hole densities by suppressing nonradiative recombination, thereby resulting in an enhanced external quantum efficiency of 8.2 %, from 1.5 % for the initial device, as well as a 2.6‐fold improved operational stability of the device under ambient conditions. Moreover, very recently, Han et al. strategically designed 2D/3D hetero‐phased structures on the surface of 3D bulk perovskite films that reduced the trap density and improved the long‐term stability of perovskite LEDs.^[122]^ The fabricated LEDs with 2D/3D hetero‐phased perovskite structures showed a higher electroluminescence efficiency of 7.70 ph per el% and an operational lifetime of 200 h compared to a pure 3D device with 0.46 ph per el% and 0.2 h, respectively.

**Table 1:** Summary of various large organic molecules for the passivation of perovskite films and their photovoltaic parameters.

Structure	Large organic molecules (LOMs)	Molecular structure	*V*_OC_ (V)	FF	*J*_SC_ (mA cm^−2^)	PCE (%)	Ref.
2D/3D mixed perovskite	BAI	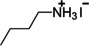	1.14	0.80	22.7	20.6	[106a]
			1.11	0.78	22.4	19.5	[108a]
	EDAI_2_	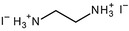	0.58	0.72	21.3	8.9	[108b]
	PEAI	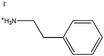	0.96	0.74	19.9	14.3	[109a]
	PEACl	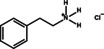	1.18	0. 79	23.6	22.0	[109b]
	4‐(aminomethyl)piperidinium (4AMP)	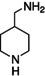	0.69	0.74	21.1	10.8	[123]
	2‐thiophenemethylammonium (ThMA)	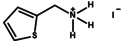	1.16	0. 81	22.8	21.4	[124]
	OAI	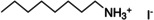	1.11	0.81	22.6	20.6	[110]
	ethane‐1,2‐diammonium	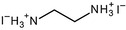	1.09	0.74	19.1	15.3	[125]
2D/3D bilayer perovskite	BAI	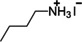	1.09	0.77	22.5	18.8	[108a]
			1.16	0.80	20.8	19.4	[113]
	PEAI	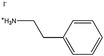	1.17	0.78	21.8	19.8	[126]
			0.813	0.79	30.3	19.4	[127]
			1.25	0.79	19.4	19.0	[117b]
			1.08	0.73	18.6	14.9	[116]
			1.07	0.73	21.3	17.0	[128]
			1.11	0.73	22.8	18.5	[129]
			1.14	0.76	22.7	20.0	[117d]
			1.04	0.68	21.1	14.9	[130]
	OAI	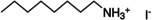	1.12	0.82	24.0	22.0	[117a]
	FPEA	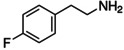	1.14	0.76	24.2	21.1	[117c]
	4‐vinylbenzylammonium (VBA) bromide	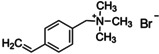	1.15	0.78	22.5	20.2	[112]
	*n*‐hexyltrimethylammonium bromide	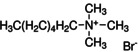	1.14	0.80	24.92	22.8	[131]
	azetidinium iodide	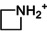	1.14	0.79	24.7	22.3	[132]
	tetraammonium zinc phthalocyanine iodide (ZnPcI)	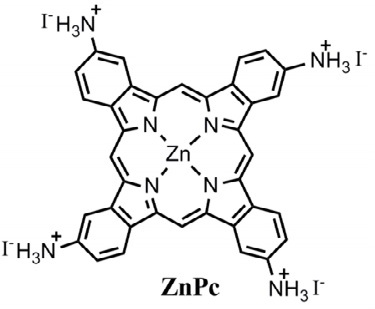	1.11	0. 77	23.5	20.2	[133]
	NH_3_I(CH_2_)_8_NH_3_I	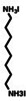	1.11	0.79	19.3	17.0	[134]
	(CF_3_)_3_CO(CH_2_)_3_NH_3_I	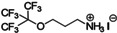	1.11	0.79	22.8	20.1	[135]
	thiazole ammonium iodide	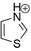	1.08	0.77	22.8	18.9	[136]
	*n*‐hexylammonium bromide (C_6_Br)	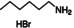	1.16	0.80	23.9	22.3	[13]
	pentafluorophenylethylammonium (FEA) iodide	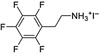	1.09	0.78	25.7	22.1	[137]
	2‐(4‐fluorophenyl)ethylammonium iodide (FPEAI)	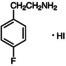	1.12	0.80	22.8	20.5	[138]
	benzylammonium iodide (BnAI)	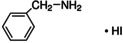	1.07	0.78	24.4	20.7	[139]

## 6 Summary and Outlook

This Review provides an in‐depth discussion to understand the types of defects in lead‐halide perovskite thin films and NCs and their influence on defect tolerance and device performance. Importantly, the halide ion dependent defect tolerance/intolerance nature of LHPs is discussed in detail. In addition, the current understanding of the surface chemistry of LHP NCs, the origin of surface defects, and passivation ligands that can be used to improve their photoluminescence efficiency and stability are highlighted. Furthermore, emerging defect passivation strategies are summarized for improving the performance of light‐emitting diodes and solar cells. Despite significant progress in understanding the surface chemistry and the development of passivation strategies for improving the optical properties of LHP NCs and thin films, there are still a number of questions that remain to be addressed to further improve the stability and efficiency of LHP optoelectronic devices.

Although the role of alkylamines in the stabilization of perovskite NCs is somewhat clear, the binding sites and the role of the alkyl acid are under debate. A few studies suggest that they bind to the halide vacancies to complete the octahedral coordination of Pb, while a few studies proposed that they form an equilibrium with the ammonium cation bound to the surface of NCs but do not directly interact with the surface of the NCs. Therefore, in‐depth studies focusing on the role of surface ligands are needed for a better understanding of their role in the surface passivation and stabilization of LHP NCs.

Cationic and anionic ligands have been used most often for the passivation; however, it is still unclear as to whether neutral ligands can bind and passivate LHP NC surfaces. Surface passivation is important for high PLQYs of NCs, while on the other hand, the surface ligands hinder the charge‐carrier transport in NC films. Therefore, it is important to find a balance between surface passivation and charge transport to improve the efficiency of the resulting optoelectronic devices.^[140]^ One alternative could be the use of small molecules or conductive organic ligand shells that could electronically couple the adjacent NCs of LHP NC films. In addition, it is important to develop ligand‐exchange strategies in both colloidal solutions and NC thin films to replace the long‐chain ligands by small molecules.

It is now broadly understood that the halide vacancies on the surfaces are the main nonradiative recombination centers in LHPs. These halide vacancies are often filled with either the corresponding halide ions after the addition of metal halides or with softer Lewis bases. The addition of metal halides could lead to the refilling of halide vacancies as well as the doping of the metal into the LHP lattice. A few studies have shown that doping metal ions into a LHP lattice can significantly improve the PLQY of CsPbCl_3_ NCs by eliminating nonradiative defects. However, it is still under debate as to whether the doping, the halide passivation, or both results in the improved PLQY of CsPbCl_3_ NCs. Moreover, the position of dopants in the LHP lattice is still unexplored. Therefore, more studies are required to decouple the roles of metal doping and halide passivation in the improvement of the optical performance and stability of LHP NCs.

To date, most studies have focused on how to eliminate identified defects in thin films and nanocrystals. There is still a lack of understanding about how defects are induced during the different synthesis methods, including precursor preparation, thin‐film crystallization, and nanocrystal purification processes. More studies are required to understand the collective mechanism of surface defect and grain boundary defect passivation. Lastly, both perovskite LEDs and solar cells suffer from operation instability because of defect evolution over time because of degradation (such as phase‐transformation or moisture‐induced degradation). Therefore, despite the need for preparing highly efficient LEDs, such as spectra‐stable blue and pure‐red LEDs, as well as high PCE solar cell devices, overcoming the limitations arising from the soft ionic nature of the material and improving the operational stability would be a key focus in the future. It should be noted that no individual strategy can passivate all defects and prohibit device instability, it is crucial to understand and utilize multiple strategies including, but not limited to, compositional engineering, device engineering, and defect passivation treatment to further improve perovskite‐based devices.

In PSCs, it is now well‐established that the structural and point defects in the polycrystalline perovskite films must be passivated to suppress nonradiative recombination and result in the simultaneous improvement of the efficiency and stability. Similar to perovskite LEDs, it has been shown that large organic molecules (LOMs) with appropriate functional groups can passivate the defect states by forming a 2D perovskite at the GBs or the surface of the 3D perovskite films, that is, a 2D/3D structure, thereby stabilizing the perovskite structure and preventing more defect formation. Different methods for obtaining the 2D/3D structures are summarized in this Review. The 2D/3D mixed and 2D/3D bilayer structures have been investigated as important passivation approaches. Whereas the 2D/3D mixed structure can be formed by adding the LOMs directly to the perovskite precursor solution, the 2D/3D bilayer structures can be formed by depositing a solution of LOMs on the top of 3D perovskite films. The state‐of‐the‐art of various 2D/3D mixed and bilayer structures have been investigated. However, we emphasize that both types of 2D/3D structures are equally important to achieve a defect‐free perovskite film. Besides the methodology, we emphasize the need to develop more efficient LOMs such as zwitterions to passivate the defect states more efficiently. Although there have been several studies of the passivation mechanism in thin films, the binding of passivating agents to the LHP thin film surface is still not well‐understood at the molecular level. More experimental and theoretical studies need to focus on the characterization of the thin‐film surfaces before and after passivation to gain insight into and to rationalize the passivation mechanism. The field of PSCs is now more mature than that of perovskite LEDs. In fact, a large variety of passivation strategies and a huge library of molecules have been reported for PSCs and it would be interesting to adapt such strategies to improve the EQEs of perovskite LEDs. We think there is a lot of synergy between perovskite LEDs and solar cells and a lot can be learnt from each other for improving their efficiencies.

## Conflict of interest

The authors declare no conflict of interest.

## Biographical Information

*Junzhi Ye received his bachelor degree in chemical and materials engineering at the University of Auckland, New Zealand. In 2019, he started PhD research in the Cavendish Laboratory, Department of Physics at the University of Cambridge, UK. His primary supervisor is Dr. Akshay Rao, and is co‐supervised by Dr. Robert Hoye. His research interests are currently focused on perovskite optoelectronic devices, including light‐emitting diodes based on perovskite quantum dots and perovskite‐silicon tandem solar cells*.

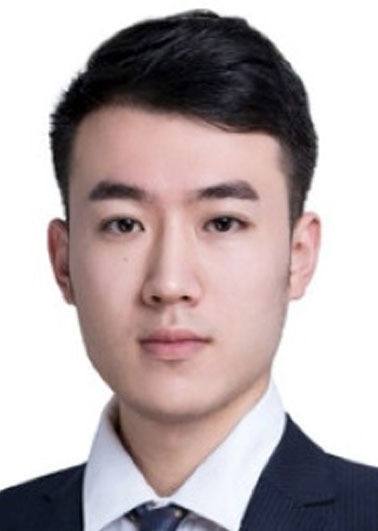

## Biographical Information

*Mahdi Malekshahi Byranvand received his PhD in inorganic chemistry at the University of Tehran in 2015. He then carried out postdoctoral research on perovskite solar cells at Karlsruhe Institute of Technology (KIT), Pohang University of Science and Technology (POSTECH), and Sharif University of technology. He is currently a researcher at the Forschungszentrum Jülich and Institute for Photovoltaics (ipv) at the University of Stuttgart in the group of Prof. Michael Saliba. His research interests focus on passivation techniques of perovskite films and perovskite‐silicon tandem solar cells*.

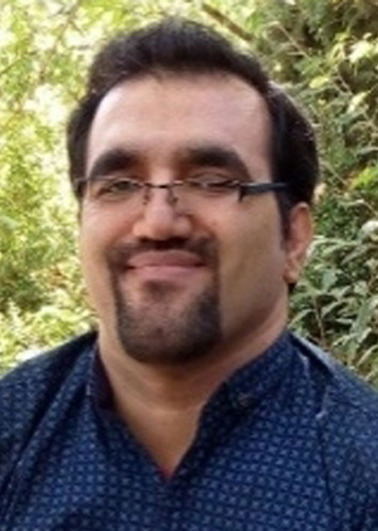

## Biographical Information

*Clara Otero received her master degree in chemical research and industrial chemistry from the University of Vigo in 2020. She is currently a PhD candidate in the group of Dr. Lakshminarayana Polavarapu at the Universisity of Vigo. Her research interests are focused on the synthesis and study of the optical properties of hybrid and inorganic halides perovskites as well as their optoelectronic applications*.

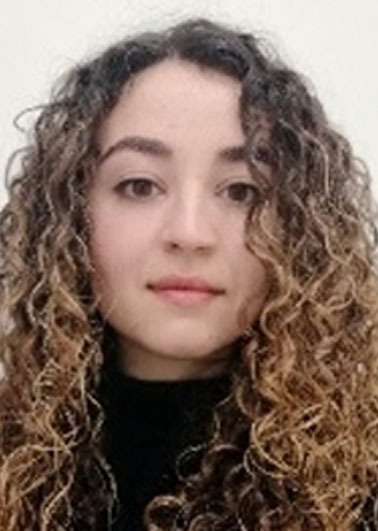

## Biographical Information

*Robert Hoye completed his PhD at the University of Cambridge (2012–2014), before working as a postdoctoral researcher at the Massachusetts Institute of Technology (2015–2016). He then received two College Research Fellowships at Cambridge (Magdalene College (2016–2019), then Downing College (2019–2020)), before taking up his Lectureship in the Department of Materials at Imperial College London in 2020. He also holds a Royal Academy of Engineering Research Fellowship. His research focusses on defect‐tolerant semiconductors, and their development into optoelectronic devices*.

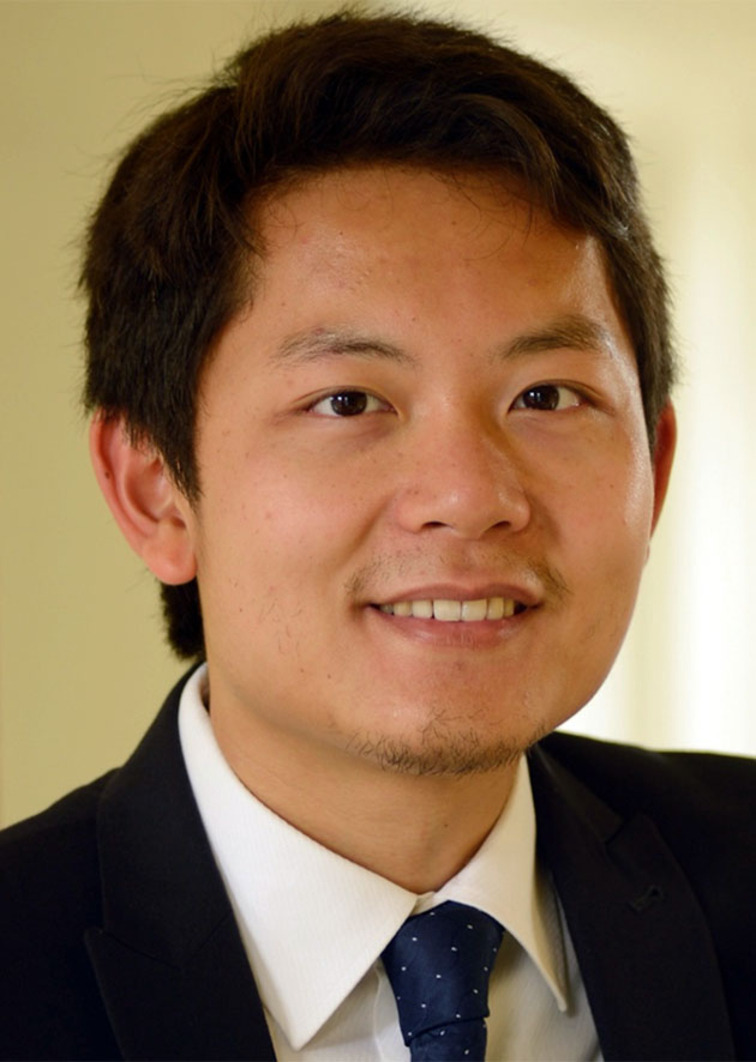

## Biographical Information

*Michael Saliba obtained his PhD at Oxford University, UK. After holding positions at TU Darmstadt, University of Fribourg, and EPFL, he became the director of the Institute for Photovoltaics (ipv) at the University of Stuttgart, with a dual appointment as the Helmholtz Young Investigator at the Forschungszentrum Jülich, Germany. His research focuses on emerging photovoltaic materials, with an emphasis on perovskites for a sustainable energy future. He has received the Heinz‐Maier‐Leibnitz Award of the German Research Foundation and was named as one of the worldwide 35 innovators under 35 by the MIT Technology Review*.

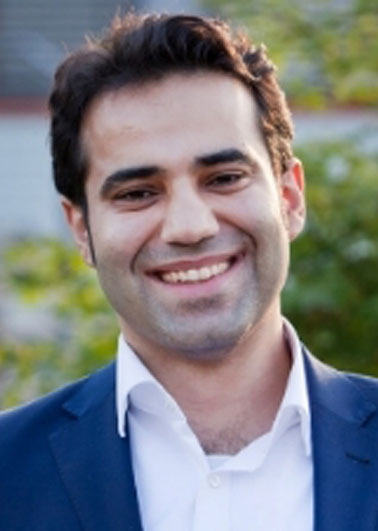

## Biographical Information

*Lakshminarayana Polavarapu obtained his MSc in Chemistry from the University of Hyderabad (India) in 2005 and PhD from the National University of Singapore in 2011. After postdoctoral research at CIC biomaGUNE and the University of Vigo in Spain, he joined the Chair for Photonics and Optoelectronics at the Ludwig‐Maximilians‐University of Munich (Germany) in 2015 as an Alexander von Humboldt postdoctoral fellow and later continued as a junior group leader. In 2020 he became the principal investigator of the Materials chemistry and physics research group at the Centro De Investigaciones Biomédicas (CINBIO), University of Vigo*.

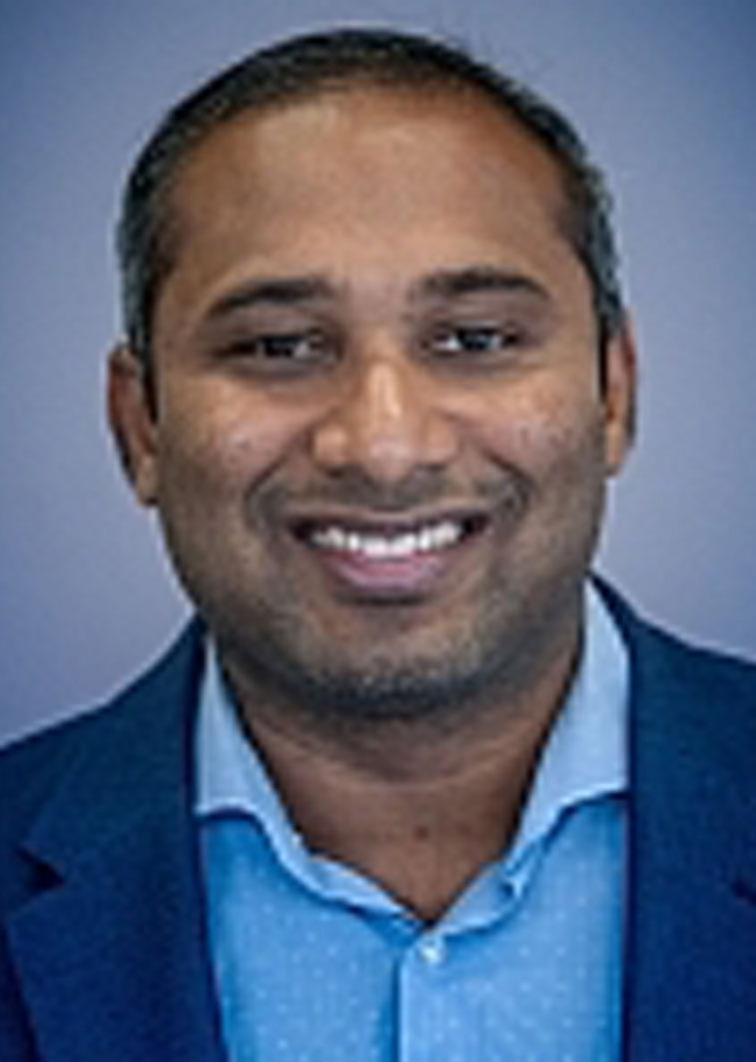

## References

[1a] H. J. Queisser , E. E. Haller , Science 1998, 281, 945–950; 9703502 10.1126/science.281.5379.945

[1b] M. D. McCluskey , A. Janotti , J. Appl. Phys. 2020, 127, 190401;

[1c] S. Mahajan , Acta Mater. 2000, 48, 137–149;

[1d] S. T. Pantelides , Rev. Mod. Phys. 1978, 50, 797–858;

[1e] H. J. von Bardeleben , D. Stiévenard , D. Deresmes , A. Huber , J. C. Bourgoin , Phys. Rev. B 1986, 34, 7192–7202; 10.1103/physrevb.34.7192 9939375

[1f] T. Goldzak , A. R. McIsaac , T. Van Voorhis , Nat. Commun. 2021, 12, 890; 33563985 10.1038/s41467-021-21153-z PMC7873310

[1g] H. J. Queisser , J. Phys. Chem. Solid. 2008, 69, 256–258.

[2a] J. Callaway , A. J. Hughes , Phys. Rev. 1967, 156, 860–876;

[2b] F. Gao , Y. Zhao , X. Zhang , J. You , Ad. Energy Mater. 2020, 10, 1902650;

[2c] J. M. Ball , A. Petrozza , Nat. Energy 2016, 1, 16149;

[2d] L. K. Ono , S. Liu , Y. Qi , Angew. Chem. Int. Ed. 2020, 59, 6676–6698; 10.1002/anie.201905521 PMC7187320 31369195

[2e] J. Bernholc , S. T. Pantelides , Phys. Rev. B 1978, 18, 1780–1789.

[3a] G. A. Baraff , M. Schlüter , Phys. Rev. Lett. 1985, 55, 1327–1330; 10031788 10.1103/PhysRevLett.55.1327

[3b] F. Greuter , G. Blatter , Semicond. Sci. Technol. 1990, 5, 111–137;

[3c] C. Wetzel , T. Suski , J. W. Ager III , E. R. Weber , E. E. Haller , S. Fischer , B. K. Meyer , R. J. Molnar , P. Perlin , Phys. Rev. Lett. 1997, 78, 3923–3926.

[4a] A. L. Vallett , S. Minassian , P. Kaszuba , S. Datta , J. M. Redwing , T. S. Mayer , Nano Lett. 2010, 10, 4813–4818; 21073180 10.1021/nl102239q

[4b] G. Zheng , W. Lu , S. Jin , C. M. Lieber , Adv. Mater. 2004, 16, 1890–1893;

[4c] S. J. Wang , M. Sawatzki , H. Kleemann , I. Lashkov , D. Wolf , A. Lubk , F. Talnack , S. Mannsfeld , Y. Krupskaya , B. Büchner , K. Leo , Mater. Today Phys. 2021, 17, 100352;

[4d] G. Gramse , A. Kölker , T. Škereň , T. J. Z. Stock , G. Aeppli , F. Kienberger , A. Fuhrer , N. J. Curson , Nat. Electronics 2020, 3, 531–538.

[5a] S.-L. Li , K. Tsukagoshi , E. Orgiu , P. Samorì , Chem. Soc. Rev. 2016, 45, 118–151; 26593874 10.1039/c5cs00517e

[5b] S. Tongay , J. Suh , C. Ataca , W. Fan , A. Luce , J. S. Kang , J. Liu , C. Ko , R. Raghunathanan , J. Zhou , F. Ogletree , J. Li , J. C. Grossman , J. Wu , Sci. Rep. 2013, 3, 2657; 24029823 10.1038/srep02657 PMC3772378

[5c] A. A. Guzelian , J. E. B. Katari , A. V. Kadavanich , U. Banin , K. Hamad , E. Juban , A. P. Alivisatos , R. H. Wolters , C. C. Arnold , J. R. Heath , J. Phys. Chem. 1996, 100, 7212–7219;

[5d] D. V. Talapin , A. L. Rogach , I. Mekis , S. Haubold , A. Kornowski , M. Haase , H. Weller , Colloid Surf. A 2002, 202, 145–154.

[6a] A. Zakutayev , C. M. Caskey , A. N. Fioretti , D. S. Ginley , J. Vidal , V. Stevanovic , E. Tea , S. Lany , J. Phys. Chem. Lett. 2014, 5, 1117–1125; 26274458 10.1021/jz5001787

[6b] R. E. Brandt , J. R. Poindexter , P. Gorai , R. C. Kurchin , R. L. Z. Hoye , L. Nienhaus , M. W. B. Wilson , J. A. Polizzotti , R. Sereika , R. Žaltauskas , L. C. Lee , J. L. MacManus-Driscoll , M. Bawendi , V. Stevanović , T. Buonassisi , Chem. Mater. 2017, 29, 4667–4674;

[6c] C. Ran , J. Xu , W. Gao , C. Huang , S. Dou , Chem. Soc. Rev. 2018, 47, 4581–4610; 29682652 10.1039/c7cs00868f

[6d] J. Kang , L.-W. Wang , J. Phys. Chem. Lett. 2017, 8, 489–493; 28071911 10.1021/acs.jpclett.6b02800

[6e] R. E. Brandt , V. Stevanović , D. S. Ginley , T. Buonassisi , MRS Commun. 2015, 5, 265–275.

[7a] M. M. Lee , J. Teuscher , T. Miyasaka , T. N. Murakami , H. J. Snaith , Science 2012, 338, 643–647; 23042296 10.1126/science.1228604

[7b] H.-S. Kim , C.-R. Lee , J.-H. Im , K.-B. Lee , T. Moehl , A. Marchioro , S.-J. Moon , R. Humphry-Baker , J.-H. Yum , J. E. Moser , M. Grätzel , N.-G. Park , Sci. Rep. 2012, 2, 591. 22912919 10.1038/srep00591 PMC3423636

[8] A. Kojima , K. Teshima , Y. Shirai , T. Miyasaka , J. Am. Chem. Soc. 2009, 131, 6050–6051. 19366264 10.1021/ja809598r

[9] J. J. Yoo , G. Seo , M. R. Chua , T. G. Park , Y. Lu , F. Rotermund , Y.-K. Kim , C. S. Moon , N. J. Jeon , J.-P. Correa-Baena , V. Bulović , S. S. Shin , M. G. Bawendi , J. Seo , Nature 2021, 590, 587–593. 33627807 10.1038/s41586-021-03285-w

[10] J. Cui , Y. Liu , Y. Deng , C. Lin , Z. Fang , C. Xiang , P. Bai , K. Du , X. Zuo , K. Wen , S. Gong , H. He , Z. Ye , Y. Gao , H. Tian , B. Zhao , J. Wang , Y. Jin , arXiv 2020, https://arxiv.org/abs/2006.07611.

[11] H. Yi-Teng , R. K. Seán , O. S. David , W. Aron , H. Robert , Nanotechnology 2020, 32, 132004.

[12a] Q. Jiang , Y. Zhao , X. Zhang , X. Yang , Y. Chen , Z. Chu , Q. Ye , X. Li , Z. Yin , J. You , Nat. Photonics 2019, 13, 460–466;

[12b] H. Jin , E. Debroye , M. Keshavarz , I. G. Scheblykin , M. B. J. Roeffaers , J. Hofkens , J. A. Steele , Mater. Horiz. 2020, 7, 397–410.

[13] J. J. Yoo , S. Wieghold , M. C. Sponseller , M. R. Chua , S. N. Bertram , N. T. P. Hartono , J. S. Tresback , E. C. Hansen , J.-P. Correa-Baena , V. Bulović , T. Buonassisi , S. S. Shin , M. G. Bawendi , Energy Environ. Sci. 2019, 12, 2192–2199.

[14a] S. ten Brinck , F. Zaccaria , I. Infante , ACS Energy Lett. 2019, 4, 2739–2747; 10.1021/acsenergylett.8b01669 PMC6333230 30662955

[14b] S. Seth , T. Ahmed , A. De , A. Samanta , ACS Energy Lett. 2019, 4, 1610–1618;

[14c] B. J. Bohn , Y. Tong , M. Gramlich , M. L. Lai , M. Döblinger , K. Wang , R. L. Z. Hoye , P. Müller-Buschbaum , S. D. Stranks , A. S. Urban , L. Polavarapu , J. Feldmann , Nano Lett. 2018, 18, 5231–5238; 29990435 10.1021/acs.nanolett.8b02190

[14d] B. A. Koscher , J. K. Swabeck , N. D. Bronstein , A. P. Alivisatos , J. Am. Chem. Soc. 2017, 139, 6566–6569. 28448140 10.1021/jacs.7b02817

[15] J. De Roo , M. Ibáñez , P. Geiregat , G. Nedelcu , W. Walravens , J. Maes , J. C. Martins , I. Van Driessche , M. V. Kovalenko , Z. Hens , ACS Nano 2016, 10, 2071–2081. 26786064 10.1021/acsnano.5b06295

[16a] A. Mahapatra , D. Prochowicz , M. M. Tavakoli , S. Trivedi , P. Kumar , P. Yadav , J. Mater. Chem. A 2020, 8, 27–54;

[16b] D. Jia , J. Chen , M. Yu , J. Liu , E. M. J. Johansson , A. Hagfeldt , X. Zhang , Small 2020, 16, 2001772. 10.1002/smll.202001772 32419275

[17a] M. I. Bodnarchuk , S. C. Boehme , S. ten Brinck , C. Bernasconi , Y. Shynkarenko , F. Krieg , R. Widmer , B. Aeschlimann , D. Günther , M. V. Kovalenko , I. Infante , ACS Energy Lett. 2019, 4, 63–74; 30662955 10.1021/acsenergylett.8b01669 PMC6333230

[17b] X. Zhu , M. Du , J. Feng , H. Wang , Z. Xu , L. Wang , S. Zuo , C. Wang , Z. Wang , C. Zhang , X. Ren , S. Priya , D. Yang , S. Liu , Angew. Chem. Int. Ed. 2021, 60, 4238–4244; 10.1002/anie.202010987 33156572

[17c] Q. Tai , X. Guo , G. Tang , P. You , T.-W. Ng , D. Shen , J. Cao , C.-K. Liu , N. Wang , Y. Zhu , C.-S. Lee , F. Yan , Angew. Chem. Int. Ed. 2019, 58, 806–810. 10.1002/anie.201811539 30499609

[18a] S. R. Smock , T. J. Williams , R. L. Brutchey , Angew. Chem. Int. Ed. 2018, 57, 11711–11715; 10.1002/anie.201806916 PMC6467082 30051545

[18b] V. K. Ravi , P. K. Santra , N. Joshi , J. Chugh , S. K. Singh , H. Rensmo , P. Ghosh , A. Nag , J. Phys. Chem. Lett. 2017, 8, 4988–4994. 28937765 10.1021/acs.jpclett.7b02192

[19a] J. Haruyama , K. Sodeyama , L. Han , Y. Tateyama , J. Phys. Chem. Lett. 2014, 5, 2903–2909; 26278097 10.1021/jz501510v

[19b] J. Haruyama , K. Sodeyama , L. Han , Y. Tateyama , Acc. Chem. Res. 2016, 49, 554–561. 26901120 10.1021/acs.accounts.5b00452

[20] I. Zimmermann , S. Aghazada , M. K. Nazeeruddin , Angew. Chem. Int. Ed. 2019, 58, 1072–1076. 10.1002/anie.201811497 30462878

[21] G. Almeida , I. Infante , L. Manna , Science 2019, 364, 833. 31147510 10.1126/science.aax5825

[22] Q. A. Akkerman , G. Rainò , M. V. Kovalenko , L. Manna , Nat. Mater. 2018, 17, 394–405. 29459748 10.1038/s41563-018-0018-4

[23] J. Kim , C.-H. Chung , K.-H. Hong , Phys. Chem. Chem. Phys. 2016, 18, 27143–27147. 27711400 10.1039/c6cp02886a

[24] S. ten Brinck , I. Infante , ACS Energy Lett. 2016, 1, 1266–1272.

[25a] E. Yassitepe , Z. Yang , O. Voznyy , Y. Kim , G. Walters , J. A. Castañeda , P. Kanjanaboos , M. Yuan , X. Gong , F. Fan , J. Pan , S. Hoogland , R. Comin , O. M. Bakr , L. A. Padilha , A. F. Nogueira , E. H. Sargent , Adv. Funct. Mater. 2016, 26, 8757–8763;

[25b] H. Lu , X. Zhu , C. Miller , J. San Martin , X. Chen , E. M. Miller , Y. Yan , M. C. Beard , J. Chem. Phys. 2019, 151, 204305. 31779317 10.1063/1.5129261

[26a] Y. Shynkarenko , M. I. Bodnarchuk , C. Bernasconi , Y. Berezovska , V. Verteletskyi , S. T. Ochsenbein , M. V. Kovalenko , ACS Energy Lett. 2019, 4, 2703–2711; 31737780 10.1021/acsenergylett.9b01915 PMC6849336

[26b] B. Zhang , L. Goldoni , J. Zito , Z. Dang , G. Almeida , F. Zaccaria , J. de Wit , I. Infante , L. De Trizio , L. Manna , Chem. Mater. 2019, 31, 9140–9147;

[26c] B. Zhang , L. Goldoni , C. Lambruschini , L. Moni , M. Imran , A. Pianetti , V. Pinchetti , S. Brovelli , L. De Trizio , L. Manna , Nano Lett. 2020, 20, 8847–8853; 33201718 10.1021/acs.nanolett.0c03833 PMC7872419

[26d] Y. Tan , Y. Zou , L. Wu , Q. Huang , D. Yang , M. Chen , M. Ban , C. Wu , T. Wu , S. Bai , T. Song , Q. Zhang , B. Sun , ACS Appl. Mater. Interfaces 2018, 10, 3784–3792; 29299911 10.1021/acsami.7b17166

[26e] J. Shamsi , D. Kubicki , M. Anaya , Y. Liu , K. Ji , K. Frohna , C. P. Grey , R. H. Friend , S. D. Stranks , ACS Energy Lett. 2020, 5, 1900–1907; 32566752 10.1021/acsenergylett.0c00935 PMC7296617

[26f] S. Wang , L. Du , Z. Jin , Y. Xin , H. Mattoussi , J. Am. Chem. Soc. 2020, 142, 12669–12680; 32588627 10.1021/jacs.0c03682

[26g] F. Krieg , S. T. Ochsenbein , S. Yakunin , S. ten Brinck , P. Aellen , A. Süess , B. Clerc , D. Guggisberg , O. Nazarenko , Y. Shynkarenko , S. Kumar , C.-J. Shih , I. Infante , M. V. Kovalenko , ACS Energy Lett. 2018, 3, 641–646; 29552638 10.1021/acsenergylett.8b00035 PMC5848145

[26h] F. Krieg , Q. K. Ong , M. Burian , G. Rainò , D. Naumenko , H. Amenitsch , A. Süess , M. J. Grotevent , F. Krumeich , M. I. Bodnarchuk , I. Shorubalko , F. Stellacci , M. V. Kovalenko , J. Am. Chem. Soc. 2019, 141, 19839–19849. 31763836 10.1021/jacs.9b09969 PMC6923794

[27] A. Walsh , A. Zunger , Nat. Mater. 2017, 16, 964–967.

[28] E. M. Tennyson , T. A. S. Doherty , S. D. Stranks , Nat. Rev. Mater. 2019, 4, 573–587.

[29] W.-J. Yin , T. Shi , Y. Yan , Appl. Phys. Lett. 2014, 104, 063903.

[30a] Y. Huang , W.-J. Yin , Y. He , J. Phys. Chem. C 2018, 122, 1345–1350;

[30b] Y. Li , C. Zhang , X. Zhang , D. Huang , Q. Shen , Y. Cheng , W. Huang , Appl. Phys. Lett. 2017, 111, 162106.

[31] D. P. Nenon , K. Pressler , J. Kang , B. A. Koscher , J. H. Olshansky , W. T. Osowiecki , M. A. Koc , L.-W. Wang , A. P. Alivisatos , J. Am. Chem. Soc. 2018, 140, 17760–17772. 30501174 10.1021/jacs.8b11035

[32] R. X. Yang , L. Z. Tan , J. Chem. Phys. 2020, 152, 034702. 31968955 10.1063/1.5128016

[33] J. Xue , R. Wang , Y. Yang , Nat. Rev. Mater. 2020, 5, 809–827.

[34] A. Buin , R. Comin , J. Xu , A. H. Ip , E. H. Sargent , Chem. Mater. 2015, 27, 4405–4412.

[35] A. Swarnkar , A. R. Marshall , E. M. Sanehira , B. D. Chernomordik , D. T. Moore , J. A. Christians , T. Chakrabarti , J. M. Luther , Science 2016, 354, 92. 27846497 10.1126/science.aag2700

[36] J. Pan , S. P. Sarmah , B. Murali , I. Dursun , W. Peng , M. R. Parida , J. Liu , L. Sinatra , N. Alyami , C. Zhao , E. Alarousu , T. K. Ng , B. S. Ooi , O. M. Bakr , O. F. Mohammed , J. Phys. Chem. Lett. 2015, 6, 5027–5033. 26624490 10.1021/acs.jpclett.5b02460

[37] J. Pan , L. N. Quan , Y. Zhao , W. Peng , B. Murali , S. P. Sarmah , M. Yuan , L. Sinatra , N. M. Alyami , J. Liu , E. Yassitepe , Z. Yang , O. Voznyy , R. Comin , M. N. Hedhili , O. F. Mohammed , Z. H. Lu , D. H. Kim , E. H. Sargent , O. M. Bakr , Adv. Mater. 2016, 28, 8718–8725. 27529532 10.1002/adma.201600784

[38a] F. Di Stasio , S. Christodoulou , N. Huo , G. Konstantatos , Chem. Mater. 2017, 29, 7663–7667;

[38b] F. Li , Y. Liu , H. Wang , Q. Zhan , Q. Liu , Z. Xia , Chem. Mater. 2018, 30, 8546–8554;

[38c] J. H. Park , A.-y. Lee , J. C. Yu , Y. S. Nam , Y. Choi , J. Park , M. H. Song , ACS Appl. Mater. Interfaces 2019, 11, 8428–8435; 30714373 10.1021/acsami.8b20808

[38d] N. Mondal , A. De , A. Samanta , ACS Energy Lett. 2019, 4, 32–39;

[38e] J. Pan , Y. Shang , J. Yin , M. De Bastiani , W. Peng , I. Dursun , L. Sinatra , A. M. El-Zohry , M. N. Hedhili , A.-H. Emwas , O. F. Mohammed , Z. Ning , O. M. Bakr , J. Am. Chem. Soc. 2018, 140, 562–565; 29249159 10.1021/jacs.7b10647

[38f] T. Ahmed , S. Seth , A. Samanta , Chem. Mater. 2018, 30, 3633–3637.

[39] G. Li , J. Huang , H. Zhu , Y. Li , J.-X. Tang , Y. Jiang , Chem. Mater. 2018, 30, 6099–6107.

[40] Y. Tong , E. Bladt , M. F. Aygüler , A. Manzi , K. Z. Milowska , V. A. Hintermayr , P. Docampo , S. Bals , A. S. Urban , L. Polavarapu , J. Feldmann , Angew. Chem. Int. Ed. 2016, 55, 13887–13892. 10.1002/anie.201605909 27690323

[41a] W. J. Mir , A. Swarnkar , A. Nag , Nanoscale 2019, 11, 4278–4286; 30806430 10.1039/c9nr00248k

[41b] Y. Tang , A. Lesage , P. Schall , J. Mater. Chem. C 2020, 8, 17139–17156;

[41c] Y. Huang , W. Luan , M. Liu , L. Turyanska , J. Mater. Chem. C 2020, 8, 2381–2387;

[41d] C.-C. Lin , S.-K. Huang , C.-E. Hsu , Y.-C. Huang , C.-Y. Wei , C.-Y. Wen , S.-S. Li , C.-W. Chen , C.-C. Chen , J. Phys. Chem. Lett. 2020, 11, 3287–3293. 32259448 10.1021/acs.jpclett.0c00443

[42] Y. Zhu , J. Zhao , G. Yang , X. Xu , G. Pan , Nanoscale 2020, 12, 7712–7719. 32211678 10.1039/d0nr01378a

[43] G. H. Ahmed , J. K. El-Demellawi , J. Yin , J. Pan , D. B. Velusamy , M. N. Hedhili , E. Alarousu , O. M. Bakr , H. N. Alshareef , O. F. Mohammed , ACS Energy Lett. 2018, 3, 2301–2307.

[44] S. Wang , Y. Wang , Y. Zhang , X. Zhang , X. Shen , X. Zhuang , P. Lu , W. W. Yu , S. V. Kershaw , A. L. Rogach , J. Phys. Chem. Lett. 2019, 10, 90–96. 30565947 10.1021/acs.jpclett.8b03750

[45] V. A. Hintermayr , A. F. Richter , F. Ehrat , M. Döblinger , W. Vanderlinden , J. A. Sichert , Y. Tong , L. Polavarapu , J. Feldmann , A. S. Urban , Adv. Mater. 2016, 28, 9478–9485. 27620530 10.1002/adma.201602897

[46] Y. Wu , C. Wei , X. Li , Y. Li , S. Qiu , W. Shen , B. Cai , Z. Sun , D. Yang , Z. Deng , H. Zeng , ACS Energy Lett. 2018, 3, 2030–2037.

[47] F. Bertolotti , G. Nedelcu , A. Vivani , A. Cervellino , N. Masciocchi , A. Guagliardi , M. V. Kovalenko , ACS Nano 2019, 13, 14294–14307. 31747248 10.1021/acsnano.9b07626 PMC6933817

[48] R. K. Behera , S. Das Adhikari , S. K. Dutta , A. Dutta , N. Pradhan , J. Phys. Chem. Lett. 2018, 9, 6884–6891. 30475626 10.1021/acs.jpclett.8b03047

[49] A. Swarnkar , W. J. Mir , A. Nag , ACS Energy Lett. 2018, 3, 286–289.

[50] Z. K. Tan , R. S. Moghaddam , M. L. Lai , P. Docampo , R. Higler , F. Deschler , M. Price , A. Sadhanala , L. M. Pazos , D. Credgington , F. Hanusch , T. Bein , H. J. Snaith , R. H. Friend , Nat. Nanotechnol. 2014, 9, 687–692. 25086602 10.1038/nnano.2014.149

[51] S. Hou , M. K. Gangishetty , Q. Quan , D. N. Congreve , Joule 2018, 2, 2421–2433.

[52a] Q. Wang , X. Wang , Z. Yang , N. Zhou , Y. Deng , J. Zhao , X. Xiao , P. Rudd , A. Moran , Y. Yan , J. Huang , Nat. Commun. 2019, 10, 5633; 31822670 10.1038/s41467-019-13580-w PMC6904584

[52b] Y. Liu , J. Cui , K. Du , H. Tian , Z. He , Q. Zhou , Z. Yang , Y. Deng , D. Chen , X. Zuo , Y. Ren , L. Wang , H. Zhu , B. Zhao , D. Di , J. Wang , R. H. Friend , Y. Jin , Nat. Photonics 2019, 13, 760–764;

[52c] Z. Li , Z. Chen , Y. Yang , Q. Xue , H.-L. Yip , Y. Cao , Nat. Commun. 2019, 10, 1027; 30833581 10.1038/s41467-019-09011-5 PMC6399279

[52d] K. Lin , J. Xing , L. N. Quan , F. P. G. de Arquer , X. Gong , J. Lu , L. Xie , W. Zhao , D. Zhang , C. Yan , W. Li , X. Liu , Y. Lu , J. Kirman , E. H. Sargent , Q. Xiong , Z. Wei , Nature 2018, 562, 245–248; 30305741 10.1038/s41586-018-0575-3

[52e] Y. Shen , M.-N. Li , Y. Li , F.-M. Xie , H.-Y. Wu , G.-H. Zhang , L. Chen , S.-T. Lee , J.-X. Tang , ACS Nano 2020, 14, 6107–6116; 32223190 10.1021/acsnano.0c01908

[52f] M.-H. Park , J. Park , J. Lee , H. S. So , H. Kim , S.-H. Jeong , T.-H. Han , C. Wolf , H. Lee , S. Yoo , T.-W. Lee , Adv. Funct. Mater. 2019, 29, 1902017;

[52g] T. Chiba , Y. Hayashi , H. Ebe , K. Hoshi , J. Sato , S. Sato , Y.-J. Pu , S. Ohisa , J. Kido , Nat. Photonics 2018, 12, 681–687;

[52h] Z. Fang , W. Chen , Y. Shi , J. Zhao , S. Chu , J. Zhang , Z. Xiao , Adv. Funct. Mater. 2020, 30, 1909754;

[52i] W. Xu , Q. Hu , S. Bai , C. Bao , Y. Miao , Z. Yuan , T. Borzda , A. J. Barker , E. Tyukalova , Z. Hu , M. Kawecki , H. Wang , Z. Yan , X. Liu , X. Shi , K. Uvdal , M. Fahlman , W. Zhang , M. Duchamp , J.-M. Liu , A. Petrozza , J. Wang , L.-M. Liu , W. Huang , F. Gao , Nat. Photonics 2019, 13, 418–424;

[52j] Y. Cao , N. Wang , H. Tian , J. Guo , Y. Wei , H. Chen , Y. Miao , W. Zou , K. Pan , Y. He , H. Cao , Y. Ke , M. Xu , Y. Wang , M. Yang , K. Du , Z. Fu , D. Kong , D. Dai , Y. Jin , G. Li , H. Li , Q. Peng , J. Wang , W. Huang , Nature 2018, 562, 249–253; 30305742 10.1038/s41586-018-0576-2

[52k] B. Zhao , S. Bai , V. Kim , R. Lamboll , R. Shivanna , F. Auras , J. M. Richter , L. Yang , L. Dai , M. Alsari , X.-J. She , L. Liang , J. Zhang , S. Lilliu , P. Gao , H. J. Snaith , J. Wang , N. C. Greenham , R. H. Friend , D. Di , Nat. Photonics 2018, 12, 783–789;

[52l] Y. Dong , Y.-K. Wang , F. Yuan , A. Johnston , Y. Liu , D. Ma , M.-J. Choi , B. Chen , M. Chekini , S.-W. Baek , L. K. Sagar , J. Fan , Y. Hou , M. Wu , S. Lee , B. Sun , S. Hoogland , R. Quintero-Bermudez , H. Ebe , P. Todorovic , F. Dinic , P. Li , H. T. Kung , M. I. Saidaminov , E. Kumacheva , E. Spiecker , L.-S. Liao , O. Voznyy , Z.-H. Lu , E. H. Sargent , Nat. Nanotechnol. 2020, 15, 668–674. 32632321 10.1038/s41565-020-0714-5

[53] S. D. Stranks , R. L. Z. Hoye , D. Di , R. H. Friend , F. Deschler , Adv. Mater. 2019, 31, 1803336. 10.1002/adma.201803336 30187974

[54a] K. Sim , T. Jun , J. Bang , H. Kamioka , J. Kim , H. Hiramatsu , H. Hosono , Appl. Phys. Rew. 2019, 6, 031402;

[54b] Q. Zhang , D. Zhang , Y. Fu , S. Poddar , L. Shu , X. Mo , Z. Fan , Adv. Funct. Mater. 2020, 30, 2002570.

[55] L. M. Herz , Annu. Rev. Phys. Chem. 2016, 67, 65–89. 26980309 10.1146/annurev-physchem-040215-112222

[56] D. Marongiu , M. Saba , F. Quochi , A. Mura , G. Bongiovanni , J. Mater. Chem. C 2019, 7, 12006–12018.

[57] J. S. Manser , P. V. Kamat , Nat. Photonics 2014, 8, 737–743.

[58] G. Xing , B. Wu , X. Wu , M. Li , B. Du , Q. Wei , J. Guo , E. K. L. Yeow , T. C. Sum , W. Huang , Nat. Commun. 2017, 8, 14558. 28239146 10.1038/ncomms14558 PMC5333353

[59] V. Sarritzu , N. Sestu , D. Marongiu , X. Chang , Q. Wang , M. A. Loi , F. Quochi , M. Saba , A. Mura , G. Bongiovanni , Adv. Opt. Mater. 2018, 6, 1700839.

[60a] M. Baranowski , P. Plochocka , Adv. Energy Mater. 2020, 10, 1903659;

[60b] M. C. Gélvez-Rueda , M. B. Fridriksson , R. K. Dubey , W. F. Jager , W. van der Stam , F. C. Grozema , Nat. Commun. 2020, 11, 1901; 32312981 10.1038/s41467-020-15869-7 PMC7171160

[60c] P. Chen , Y. Bai , M. Lyu , J.-H. Yun , M. Hao , L. Wang , Solar RRL 2018, 2, 1700186.

[61] N. Kawano , M. Koshimizu , K. Asai , J. Phys. Chem. C 2012, 116, 22992–22995.

[62] P. Papagiorgis , D. Tsokkou , K. Gahlot , L. Protesescu , A. Manoli , F. Hermerschmidt , C. Christodoulou , S. A. Choulis , M. V. Kovalenko , A. Othonos , G. Itskos , J. Phys. Chem. C 2020, 124, 27848–27857.

[63] A. Rossi , M. B. Price , J. Hardy , J. Gorman , T. W. Schmidt , N. J. L. K. Davis , J. Phys. Chem. C 2020, 124, 3306–3313.

[64a] J. Chang , Y. Ogomi , C. Ding , Y. H. Zhang , T. Toyoda , S. Hayase , K. Katayama , Q. Shen , Phys. Chem. Chem. Phys. 2017, 19, 6358–6367; 27901148 10.1039/c6cp06561a

[64b] J. J. Grenland , C. J. A. Maddux , D. F. Kelley , A. M. Kelley , J. Phys. Chem. Lett. 2017, 8, 5113–5118; 28972776 10.1021/acs.jpclett.7b02242

[64c] M. Micheel , B. Liu , M. Wächtler , Catalysts 2020, 10, 1143.

[65] M. Hao , Y. Bai , S. Zeiske , L. Ren , J. Liu , Y. Yuan , N. Zarrabi , N. Cheng , M. Ghasemi , P. Chen , M. Lyu , D. He , J.-H. Yun , Y. Du , Y. Wang , S. Ding , A. Armin , P. Meredith , G. Liu , H.-M. Cheng , L. Wang , Nat. Energy 2020, 5, 79–88.

[66] X.-K. Liu , W. Xu , S. Bai , Y. Jin , J. Wang , R. H. Friend , F. Gao , Nat. Mater 2021, 20, 10–21. 32929252 10.1038/s41563-020-0784-7

[67a] D. Luo , Q. Chen , Y. Qiu , M. Zhang , B. Liu , Nanomaterials 2019, 9; 10.3390/nano9071007 PMC6669542 31336905

[67b] E. Yoon , K. Y. Jang , J. Park , T.-W. Lee , Adv. Mater. Interfaces 2021, 8, 2001712.

[68a] R. L. Z. Hoye , M.-L. Lai , M. Anaya , Y. Tong , K. Gałkowski , T. Doherty , W. Li , T. N. Huq , S. Mackowski , L. Polavarapu , J. Feldmann , J. L. MacManus-Driscoll , R. H. Friend , A. S. Urban , S. D. Stranks , ACS Energy Lett. 2019, 4, 1181–1188; 31119197 10.1021/acsenergylett.9b00571 PMC6516044

[68b] X. Zhang , H. Lin , H. Huang , C. Reckmeier , Y. Zhang , W. C. H. Choy , A. L. Rogach , Nano Lett. 2016, 16, 1415–1420. 26745020 10.1021/acs.nanolett.5b04959

[69a] L. Lan , B. Liu , H. Tao , J. Zou , C. Jiang , M. Xu , L. Wang , J. Peng , Y. Cao , J. Mater. Chem. C 2019, 7, 5755–5763;

[69b] D. B. Kim , J. C. Yu , Y. S. Nam , D. W. Kim , E. D. Jung , S. Y. Lee , S. Lee , J. H. Park , A.-Y. Lee , B. R. Lee , D. Di Nuzzo , R. H. Friend , M. H. Song , J. Mater. Chem. C 2016, 4, 8161–8165;

[69c] X. Shen , H. Wu , X. Zhang , M. Xu , J. Hu , J. Zhu , B. Dong , W. W. Yu , X. Bai , J. Phys. Chem. Lett. 2021, 12, 94–100. 33306374 10.1021/acs.jpclett.0c03047

[70] Y. Shi , W. Wu , H. Dong , G. Li , K. Xi , G. Divitini , C. Ran , F. Yuan , M. Zhang , B. Jiao , X. Hou , Z. Wu , Adv. Mater. 2018, 30, 1800251. 10.1002/adma.201800251 29733472

[71] B. Rivkin , P. Fassl , Q. Sun , A. D. Taylor , Z. Chen , Y. Vaynzof , ACS Omega 2018, 3, 10042–10047. 31459132 10.1021/acsomega.8b01626 PMC6644495

[72a] Q. Dong , L. Lei , J. Mendes , F. So , J. Phys. Mater. 2020, 3, 012002;

[72b] R. Wang , M. Mujahid , Y. Duan , Z.-K. Wang , J. Xue , Y. Yang , Adv. Funct. Mater. 2019, 29, 1808843.

[73] X. Ren , Z. Wang , W. E. I. Sha , W. C. H. Choy , ACS Photonics 2017, 4, 934–942.

[74] M. Sajedi Alvar , P. W. M. Blom , G.-J. A. H. Wetzelaer , Nat. Commun. 2020, 11, 4023. 32782256 10.1038/s41467-020-17868-0 PMC7419305

[75] I. Kim , P. Kivisaari , J. Oksanen , S. Suihkonen , Materials 2017, 10, 1421. 10.3390/ma10121421 PMC5744356 29231900

[76] E. Bi , H. Chen , F. Xie , Y. Wu , W. Chen , Y. Su , A. Islam , M. Grätzel , X. Yang , L. Han , Nat. Commun. 2017, 8, 15330. 28604673 10.1038/ncomms15330 PMC5472713

[77] Y.-H. Kim , S. Kim , A. Kakekhani , J. Park , J. Park , Y.-H. Lee , H. Xu , S. Nagane , R. B. Wexler , D.-H. Kim , S. H. Jo , L. Martínez-Sarti , P. Tan , A. Sadhanala , G.-S. Park , Y.-W. Kim , B. Hu , H. J. Bolink , S. Yoo , R. H. Friend , A. M. Rappe , T.-W. Lee , Nat. Photonics 2021, 15, 148–155.

[78a] S. Zhang , H. Liu , X. Li , S. Wang , Nano Energy 2020, 77, 105302;

[78b] H.-C. Wang , W. Wang , A.-C. Tang , H.-Y. Tsai , Z. Bao , T. Ihara , N. Yarita , H. Tahara , Y. Kanemitsu , S. Chen , R.-S. Liu , Angew. Chem. Int. Ed. 2017, 56, 13650–13654. 10.1002/anie.201706860 28865137

[79] M. Lu , X. Zhang , X. Bai , H. Wu , X. Shen , Y. Zhang , W. Zhang , W. Zheng , H. Song , W. W. Yu , A. L. Rogach , ACS Energy Lett. 2018, 3, 1571–1577. 30505950 10.1021/acsenergylett.8b00835 PMC6269143

[80] K. Sivalertporn , L. Mouchliadis , A. L. Ivanov , R. Philp , E. A. Muljarov , Phys. Rev. B 2012, 85, 045207.

[81] S. Zou , Y. Liu , J. Li , C. Liu , R. Feng , F. Jiang , Y. Li , J. Song , H. Zeng , M. Hong , X. Chen , J. Am. Chem. Soc. 2017, 139, 11443–11450. 28756676 10.1021/jacs.7b04000

[82] M. Lu , X. Zhang , Y. Zhang , J. Guo , X. Shen , W. W. Yu , A. L. Rogach , Adv. Mater. 2018, 30, 1804691. 10.1002/adma.201804691 30306648

[83] X. Shen , Y. Zhang , S. V. Kershaw , T. Li , C. Wang , X. Zhang , W. Wang , D. Li , Y. Wang , M. Lu , L. Zhang , C. Sun , D. Zhao , G. Qin , X. Bai , W. W. Yu , A. L. Rogach , Nano Lett. 2019, 19, 1552–1559. 30741555 10.1021/acs.nanolett.8b04339

[84] S. Lee , J. H. Park , B. R. Lee , E. D. Jung , J. C. Yu , D. Di Nuzzo , R. H. Friend , M. H. Song , J. Phys. Chem. Lett. 2017, 8, 1784–1792. 28378585 10.1021/acs.jpclett.7b00372

[85] J. N. Yang , Y. Song , J. S. Yao , K. H. Wang , J. J. Wang , B. S. Zhu , M. M. Yao , S. U. Rahman , Y. F. Lan , F. J. Fan , H. B. Yao , J. Am. Chem. Soc. 2020, 142, 2956–2967. 31902206 10.1021/jacs.9b11719

[86] S. Gonzalez-Carrero , L. C. Schmidt , I. Rosa-Pardo , L. Martínez-Sarti , M. Sessolo , R. E. Galian , J. Pérez-Prieto , ACS Omega 2018, 3, 1298–1303. 31457965 10.1021/acsomega.7b02052 PMC6641344

[87a] D. Ma , P. Todorović , S. Meshkat , M. I. Saidaminov , Y.-K. Wang , B. Chen , P. Li , B. Scheffel , R. Quintero-Bermudez , J. Z. Fan , Y. Dong , B. Sun , C. Xu , C. Zhou , Y. Hou , X. Li , Y. Kang , O. Voznyy , Z.-H. Lu , D. Ban , E. H. Sargent , J. Am. Chem. Soc. 2020, 142, 5126–5134; 32150404 10.1021/jacs.9b12323

[87b] H. Zhang , F. Ye , W. Li , J. Yao , R. S. Gurney , D. Liu , C. Xiong , T. Wang , Org. Electronics 2019, 67, 187–193.

[88a] W. Yin , M. Li , W. Dong , Z. Luo , Y. Li , J. Qian , J. Zhang , W. Zhang , Y. Zhang , S. V. Kershaw , X. Zhang , W. Zheng , A. L. Rogach , ACS Energy Lett. 2021, 6, 477–484;

[88b] M. Lu , J. Guo , S. Sun , P. Lu , J. Wu , Y. Wang , S. V. Kershaw , W. W. Yu , A. L. Rogach , Y. Zhang , Nano Lett. 2020, 20, 2829–2836. 32223199 10.1021/acs.nanolett.0c00545

[89] M. Karlsson , Z. Yi , S. Reichert , X. Luo , W. Lin , Z. Zhang , C. Bao , R. Zhang , S. Bai , G. Zheng , P. Teng , L. Duan , Y. Lu , K. Zheng , T. Pullerits , C. Deibel , W. Xu , R. Friend , F. Gao , Nat. Commun. 2021, 12, 361. 33441549 10.1038/s41467-020-20582-6 PMC7806600

[90] Z. Ren , L. Li , J. Yu , R. Ma , X. Xiao , R. Chen , K. Wang , X. W. Sun , W.-J. Yin , W. C. H. Choy , ACS Energy Lett. 2020, 5, 2569–2579.

[91a] Y. Wang , J. He , H. Chen , J. Chen , R. Zhu , P. Ma , A. Towers , Y. Lin , A. J. Gesquiere , S.-T. Wu , Y. Dong , Adv. Mater. 2016, 28, 10710–10717; 27748549 10.1002/adma.201603964

[91b] W. Cai , Z. Chen , Z. Li , L. Yan , D. Zhang , L. Liu , Q.-h. Xu , Y. Ma , F. Huang , H.-L. Yip , Y. Cao , ACS Appl. Mater. Interfaces 2018, 10, 42564–42572. 30403343 10.1021/acsami.8b13418

[92] L. Xu , J. Li , B. Cai , J. Song , F. Zhang , T. Fang , H. Zeng , Nat. Commun. 2020, 11, 3902. 32764550 10.1038/s41467-020-17633-3 PMC7413529

[93] S. Chen , Y. Liu , X. Xiao , Z. Yu , Y. Deng , X. Dai , Z. Ni , J. Huang , Joule 2020, 4, 2661–2674.

[94] J. C. Yu , J. H. Park , S. Y. Lee , M. H. Song , Nanoscale 2019, 11, 1505–1514. 30643913 10.1039/c8nr08683d

[95] S. Kumar , J. Jagielski , T. Marcato , S. F. Solari , C.-J. Shih , J. Phys. Chem. Lett. 2019, 10, 7560–7567. 31736317 10.1021/acs.jpclett.9b02950 PMC6926956

[96] C. Liu , Q. Zeng , Y. Zhao , Y. Yu , M. Yang , H. Gao , H. Wei , B. Yang , Solar RRL 2020, 4, 2000102.

[97] J. Qin , J. Zhang , Y. Bai , S. Ma , M. Wang , H. Xu , M. Loyd , Y. Zhan , X. Hou , B. Hu , iScience 2019, 19, 378–387. 31419631 10.1016/j.isci.2019.07.044 PMC6706605

[98] A. Maiti , S. Chatterjee , L. Peedikakkandy , A. J. Pal , Solar RRL 2020, 4, 2000505.

[99a] L. Fu , H. Li , L. Wang , R. Yin , B. Li , L. Yin , Energy Environ. Sci. 2020, 13, 4017–4056;

[99b] H. Lu , A. Krishna , S. M. Zakeeruddin , M. Grätzel , A. Hagfeldt , iScience 2020, 23, 101359; 32712463 10.1016/j.isci.2020.101359 PMC7390817

[99c] Y. Zhou , W. Chen , J. Appl. Phys. 2020, 128, 200401.

[100a] W. Tress , N. Marinova , T. Moehl , S. M. Zakeeruddin , M. K. Nazeeruddin , M. Grätzel , Energy Environ. Sci. 2015, 8, 995–1004;

[100b] B. Chen , P. N. Rudd , S. Yang , Y. Yuan , J. Huang , Chem. Soc. Rev. 2019, 48, 3842–3867. 31187791 10.1039/c8cs00853a

[101] N. Ahn , K. Kwak , M. S. Jang , H. Yoon , B. Y. Lee , J.-K. Lee , P. V. Pikhitsa , J. Byun , M. Choi , Nat. Commun. 2016, 7, 13422. 27830709 10.1038/ncomms13422 PMC5110646

[102a] D. J. Kubicki , D. Prochowicz , A. Hofstetter , M. Saski , P. Yadav , D. Bi , N. Pellet , J. Lewiński , S. M. Zakeeruddin , M. Grätzel , L. Emsley , J. Am. Chem. Soc. 2018, 140, 3345–3351; 29429335 10.1021/jacs.7b12860

[102b] H. Min , M. Kim , S.-U. Lee , H. Kim , G. Kim , K. Choi , J. H. Lee , S. I. Seok , Science 2019, 366, 749. 31699938 10.1126/science.aay7044

[103a] M. Saliba , T. Matsui , J.-Y. Seo , K. Domanski , J.-P. Correa-Baena , M. K. Nazeeruddin , S. M. Zakeeruddin , W. Tress , A. Abate , A. Hagfeldt , M. Grätzel , Energy Environ. Sci. 2016, 9, 1989–1997; 27478500 10.1039/c5ee03874j PMC4936376

[103b] J.-P. Correa-Baena , M. Saliba , T. Buonassisi , M. Grätzel , A. Abate , W. Tress , A. Hagfeldt , Science 2017, 358, 739. 29123060 10.1126/science.aam6323

[104a] G. Grancini , M. K. Nazeeruddin , Nat. Rev. Mater. 2019, 4, 4–22;

[104b] A. Krishna , S. Gottis , M. K. Nazeeruddin , F. Sauvage , Adv. Funct. Mater. 2019, 29, 1806482.

[105] H. Tsai , W. Nie , J.-C. Blancon , C. C. Stoumpos , R. Asadpour , B. Harutyunyan , A. J. Neukirch , R. Verduzco , J. J. Crochet , S. Tretiak , L. Pedesseau , J. Even , M. A. Alam , G. Gupta , J. Lou , P. M. Ajayan , M. J. Bedzyk , M. G. Kanatzidis , A. D. Mohite , Nature 2016, 536, 312–316. 27383783 10.1038/nature18306

[106a] Z. Wang , Q. Lin , F. P. Chmiel , N. Sakai , L. M. Herz , H. J. Snaith , Nat. Energy 2017, 2, 17135;

[106b] G. Grancini , C. Roldán-Carmona , I. Zimmermann , E. Mosconi , X. Lee , D. Martineau , S. Narbey , F. Oswald , F. De Angelis , M. Graetzel , M. K. Nazeeruddin , Nat. Commun. 2017, 8, 15684. 28569749 10.1038/ncomms15684 PMC5461484

[107] T. Niu , J. Lu , M.-C. Tang , D. Barrit , D.-M. Smilgies , Z. Yang , J. Li , Y. Fan , T. Luo , I. McCulloch , A. Amassian , S. Liu , K. Zhao , Energy Environ. Sci. 2018, 11, 3358–3366.

[108a] Y. Lin , Y. Bai , Y. Fang , Z. Chen , S. Yang , X. Zheng , S. Tang , Y. Liu , J. Zhao , J. Huang , J. Phys. Chem. Lett. 2018, 9, 654–658; 29350044 10.1021/acs.jpclett.7b02679

[108b] E. Jokar , C.-H. Chien , A. Fathi , M. Rameez , Y.-H. Chang , E. W.-G. Diau , Energy Environ. Sci. 2018, 11, 2353–2362.

[109a] D. S. Lee , J. S. Yun , J. Kim , A. M. Soufiani , S. Chen , Y. Cho , X. Deng , J. Seidel , S. Lim , S. Huang , A. W. Y. Ho-Baillie , ACS Energy Lett. 2018, 3, 647–654;

[109b] W.-Q. Wu , P. N. Rudd , Q. Wang , Z. Yang , J. Huang , Adv. Mater. 2020, 32, 2000995; 10.1002/adma.202000995 32468688

[109c] J. Shi , Y. Gao , X. Gao , Y. Zhang , J. Zhang , X. Jing , M. Shao , Adv. Mater. 2019, 31, 1901673. 10.1002/adma.201901673 31379023

[110] M. Jung , T. J. Shin , J. Seo , G. Kim , S. I. Seok , Energy Environ. Sci. 2018, 11, 2188–2197.

[111] T. Zhang , L. Xie , L. Chen , N. Guo , G. Li , Z. Tian , B. Mao , Y. Zhao , Adv. Funct. Mater. 2017, 27, 1603568.

[112] A. H. Proppe , M. Wei , B. Chen , R. Quintero-Bermudez , S. O. Kelley , E. H. Sargent , J. Am. Chem. Soc. 2019, 141, 14180–14189. 31422664 10.1021/jacs.9b05083

[113] Z. Liu , K. Meng , X. Wang , Z. Qiao , Q. Xu , S. Li , L. Cheng , Z. Li , G. Chen , Nano Lett. 2020, 20, 1296–1304. 31986053 10.1021/acs.nanolett.9b04759

[114] X. Zheng , Y. Hou , C. Bao , J. Yin , F. Yuan , Z. Huang , K. Song , J. Liu , J. Troughton , N. Gasparini , C. Zhou , Y. Lin , D.-J. Xue , B. Chen , A. K. Johnston , N. Wei , M. N. Hedhili , M. Wei , A. Y. Alsalloum , P. Maity , B. Turedi , C. Yang , D. Baran , T. D. Anthopoulos , Y. Han , Z.-H. Lu , O. F. Mohammed , F. Gao , E. H. Sargent , O. M. Bakr , Nat. Energy 2020, 5, 131–140.

[115] D. H. Kim , C. P. Muzzillo , J. Tong , A. F. Palmstrom , B. W. Larson , C. Choi , S. P. Harvey , S. Glynn , J. B. Whitaker , F. Zhang , Z. Li , H. Lu , M. F. A. M. van Hest , J. J. Berry , L. M. Mansfield , Y. Huang , Y. Yan , K. Zhu , Joule 2019, 3, 1734–1745.

[116] Y. Hu , J. Schlipf , M. Wussler , M. L. Petrus , W. Jaegermann , T. Bein , P. Müller-Buschbaum , P. Docampo , ACS Nano 2016, 10, 5999–6007. 27228558 10.1021/acsnano.6b01535

[117a] H. Kim , S.-U. Lee , D. Y. Lee , M. J. Paik , H. Na , J. Lee , S. I. Seok , Adv. Energy Mater. 2019, 9, 1970187;

[117b] C. Chen , Z. Song , C. Xiao , R. A. Awni , C. Yao , N. Shrestha , C. Li , S. S. Bista , Y. Zhang , L. Chen , R. J. Ellingson , C.-S. Jiang , M. Al-Jassim , G. Fang , Y. Yan , ACS Energy Lett. 2020, 5, 2560–2568;

[117c] T. Niu , J. Lu , X. Jia , Z. Xu , M.-C. Tang , D. Barrit , N. Yuan , J. Ding , X. Zhang , Y. Fan , T. Luo , Y. Zhang , D.-M. Smilgies , Z. Liu , A. Amassian , S. Jin , K. Zhao , S. Liu , Nano Lett. 2019, 19, 7181–7190; 31479275 10.1021/acs.nanolett.9b02781

[117d] K. T. Cho , G. Grancini , Y. Lee , E. Oveisi , J. Ryu , O. Almora , M. Tschumi , P. A. Schouwink , G. Seo , S. Heo , J. Park , J. Jang , S. Paek , G. Garcia-Belmonte , M. K. Nazeeruddin , Energy Environ. Sci. 2018, 11, 952–959.

[118] J. Chen , J.-Y. Seo , N.-G. Park , Adv. Energy Mater. 2018, 8, 1702714.

[119] Y. Zheng , R. Naphade , N. Mondal , O. M. Bakr , O. F. Mohammed , Y. N. Gartstein , A. V. Malko , ACS Photonics 2021, 8, 276–282.

[120a] S. Heo , G. Seo , K. T. Cho , Y. Lee , S. Paek , S. Kim , M. Seol , S. H. Kim , D.-J. Yun , K. Kim , J. Park , J. Lee , L. Lechner , T. Rodgers , J. W. Chung , J.-S. Kim , D. Lee , S.-H. Choi , M. K. Nazeeruddin , Adv. Energy Mater. 2019, 9, 1902470;

[120b] W. Cha , H. Han , Y. Hong , G. Kim , C. Park , D. Kim , J. Phys. Chem. C 2020, 124, 4414–4420.

[121] F. Zhang , B. Cai , J. Song , B. Han , B. Zhang , H. Zeng , Adv. Funct. Mater. 2020, 30, 2001732.

[122] T.-H. Han , J.-W. Lee , Y. J. Choi , C. Choi , S. Tan , S.-J. Lee , Y. Zhao , Y. Huang , D. Kim , Y. Yang , Adv. Mater. 2020, 32, 1905674. 10.1002/adma.201905674 31737948

[123] M. Chen , Q. Dong , F. T. Eickemeyer , Y. Liu , Z. Dai , A. D. Carl , B. Bahrami , A. H. Chowdhury , R. L. Grimm , Y. Shi , Q. Qiao , S. M. Zakeeruddin , M. Grätzel , N. P. Padture , ACS Energy Lett. 2020, 5, 2223–2230.

[124] T. Zhou , H. Lai , T. Liu , D. Lu , X. Wan , X. Zhang , Y. Liu , Y. Chen , Adv. Mater. 2019, 31, 1901242. 10.1002/adma.201901242 31194267

[125] J. Cho , S. Banerjee , Chem. Mater. 2018, 30, 6144–6155.

[126] Y. Bai , S. Xiao , C. Hu , T. Zhang , X. Meng , H. Lin , Y. Yang , S. Yang , Adv. Energy Mater. 2017, 7, 1701038.

[127] M. Wei , K. Xiao , G. Walters , R. Lin , Y. Zhao , M. I. Saidaminov , P. Todorović , A. Johnston , Z. Huang , H. Chen , A. Li , J. Zhu , Z. Yang , Y.-K. Wang , A. H. Proppe , S. O. Kelley , Y. Hou , O. Voznyy , H. Tan , E. H. Sargent , Adv. Mater. 2020, 32, 1907058. 10.1002/adma.201907058 32030824

[128] N. Li , Z. Zhu , Q. Dong , J. Li , Z. Yang , C.-C. Chueh , A. K. Y. Jen , L. Wang , Adv. Mater. Interfaces 2017, 4, 1700598.

[129] P. Chen , Y. Bai , S. Wang , M. Lyu , J.-H. Yun , L. Wang , Adv. Funct. Mater. 2018, 28, 1706923.

[130] K. Lee , J. Kim , H. Yu , J. W. Lee , C.-M. Yoon , S. K. Kim , J. Jang , J. Mater. Chem. A 2018, 6, 24560–24568.

[131] E. H. Jung , N. J. Jeon , E. Y. Park , C. S. Moon , T. J. Shin , T.-Y. Yang , J. H. Noh , J. Seo , Nature 2019, 567, 511–515. 30918371 10.1038/s41586-019-1036-3

[132] F. Ansari , E. Shirzadi , M. Salavati-Niasari , T. LaGrange , K. Nonomura , J.-H. Yum , K. Sivula , S. M. Zakeeruddin , M. K. Nazeeruddin , M. Grätzel , P. J. Dyson , A. Hagfeldt , J. Am. Chem. Soc. 2020, 142, 11428–11433. 32391696 10.1021/jacs.0c01704

[133] J. Cao , C. Li , X. Lv , X. Feng , R. Meng , Y. Wu , Y. Tang , J. Am. Chem. Soc. 2018, 140, 11577–11580. 30125479 10.1021/jacs.8b07025

[134] T. Zhao , C.-C. Chueh , Q. Chen , A. Rajagopal , A. K. Y. Jen , ACS Energy Lett. 2016, 1, 757–763.

[135] K. T. Cho , Y. Zhang , S. Orlandi , M. Cavazzini , I. Zimmermann , A. Lesch , N. Tabet , G. Pozzi , G. Grancini , M. K. Nazeeruddin , Nano Lett. 2018, 18, 5467–5474. 30134112 10.1021/acs.nanolett.8b01863

[136] L. Gao , I. Spanopoulos , W. Ke , S. Huang , I. Hadar , L. Chen , X. Li , G. Yang , M. G. Kanatzidis , ACS Energy Lett. 2019, 4, 1763–1769.

[137] Y. Liu , S. Akin , L. Pan , R. Uchida , N. Arora , J. V. Milić , A. Hinderhofer , F. Schreiber , A. R. Uhl , S. M. Zakeeruddin , A. Hagfeldt , M. I. Dar , M. Grätzel , Sci. Adv. 2019, 5, eaaw2543. 31187060 10.1126/sciadv.aaw2543 PMC6555633

[138] Q. Zhou , L. Liang , J. Hu , B. Cao , L. Yang , T. Wu , X. Li , B. Zhang , P. Gao , Adv. Energy Mater. 2019, 9, 1802595.

[139] H. Kim , M. Pei , Y. Lee , A. A. Sutanto , S. Paek , V. I. E. Queloz , A. J. Huckaba , K. T. Cho , H. J. Yun , H. Yang , M. K. Nazeeruddin , Adv. Funct. Mater. 2020, 30, 1910620.

[140a] N. K. Kumawat , A. Swarnkar , A. Nag , D. Kabra , J. Phys. Chem. C 2018, 122, 13767–13773;

[140b] S. Cho , J. Kim , S. M. Jeong , M. J. Ko , J.-S. Lee , Y. Kim , Chem. Mater. 2020, 32, 8808–8818;

[140c] B.-B. Zhang , S. Yuan , J.-P. Ma , Y. Zhou , J. Hou , X. Chen , W. Zheng , H. Shen , X.-C. Wang , B. Sun , O. M. Bakr , L.-S. Liao , H.-T. Sun , J. Am. Chem. Soc. 2019, 141, 15423–15432; 31469556 10.1021/jacs.9b08140

[140d] K. M. Sim , A. Swarnkar , A. Nag , D. S. Chung , Laser Photonics Rev. 2018, 12, 1700209.

